# Gut Microbiome–Sphingolipid Metabolism–Brain Axis Interactions: Neuroprotective Effects of Amitriptyline as Functional Inhibitor of Acid Sphingomyelinase in a Mouse Model of Tauopathy

**DOI:** 10.1007/s11481-025-10270-x

**Published:** 2026-01-03

**Authors:** Mennatallah Ibrahim, Asmaa M. Khalil, Heba Attia, Saleh Alseekh, Ahmed F. Mohamed, Mohammed F. EL-Yamany

**Affiliations:** 1 Postgraduate program in Pharmacology and Toxicology, Faculty of Pharmacy, Cairo University, Cairo, 11562 Egypt; 2 Department of Pharmacology and Biochemistry, Faculty of Pharmacy, Horus University, New Damietta, Egypt; 3 Department of Pharmacognosy, Faculty of Pharmacy, Cairo University, Cairo, 11562 Egypt; 4 Department of Microbiology and Immunology, Faculty of Pharmacy, Cairo University, Cairo, 11562 Egypt; 5 Center for Genome and Microbiome Research, Cairo University, Cairo, 11562 Egypt; 6 Max Planck Institute of Molecular Plant Physiology, Potsdam-Golm, Germany; 7 Department of Pharmacology and Toxicology, Faculty of Pharmacy, Cairo University, Cairo, 11562 Egypt; 8 Faculty of Pharmacy, King Salman International University (KSIU), South Sinai, 46612 Egypt

**Keywords:** Amitriptyline, Tauopathy, P301S, Acid sphingomyelinase, Gut microbiota, Sphingolipids

## Abstract

Tauopathies are neurodegenerative diseases characterized by accumulation of hyperphosphorylated tau protein (P-tau). The gut microbiota (GM) is symbiotic with the host and altered in neurodegenerative diseases. Amitriptyline (AMI) is a functional inhibitor of acid sphingomyelinase (ASM) which is abnormally highly expressed in brains of Alzheimer patients. Little data is known about the role of colonic ASM in management of tauopathy. Therefore, the aim of this study was to investigate the role of AMI on reversing gut dysbiosis, ceramide levels, colonic inflammation and intestinal barrier disruption in tauopathy through the bidirectional gut-brain axis. P301S transgenic mice were administered AMI for 35 days. Colonic ASM, ceramides, inflammation and membrane integrity were assessed besides fecal microbiome analysis and serum lipopolysaccharides to assess intestinal membrane disruption. Levels of hippocampal P-tau, protein phosphatase 2 A and neurogenesis were assessed along with cognitive behavior. AMI treatment significantly reduced colonic ASM, ceramide levels, increased abundance of *Harryflintia*, *Dubosiella*, and *Parasutterella* and decreased abundance of *Lactobacillus*, *Lachnoclostridium*, *Oscillibacter*, *Oscillospiracea UCG-003*,* Colidextribacter*, *Roseburia*,* Butyricicoccus*, and Sphingomondales. In contrast, P301S mice displayed an altered GM profile with enriched Firmicutes and Clostridia, and low proportions of Bacteroidota- a phylum associated with intestinal barrier protection-, and *Ruminococcaceae*. Also, AMI treatment decreased inflammation and restored colonic membrane integrity with subsequent decrease in serum lipopolysaccharides, P-tau in hippocampus and improvement in cognitive behaviour and neurogenesis. The current results indicate that AMI has neuroprotective effects against tauopathy through modulation of ASM activity, associated ceramide levels, GM composition, colonic inflammation and membrane integrity through bidirectional gut-brain axis.

**Graphical Abstract:**

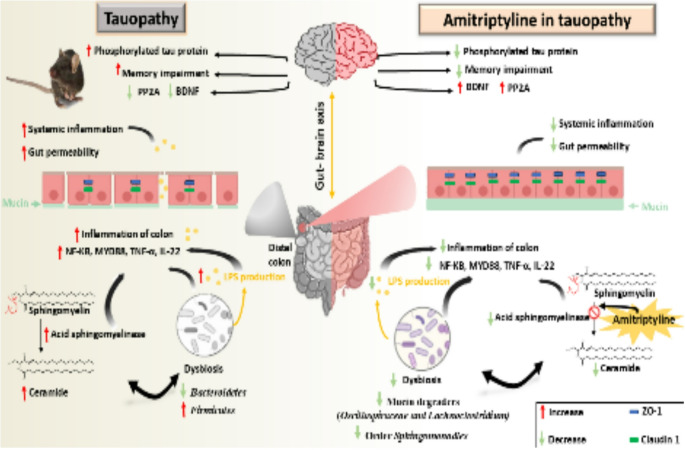

**Supplementary Information:**

The online version contains supplementary material available at 10.1007/s11481-025-10270-x.

## Introduction

Tauopathies are neurodegenerative diseases which are pathologically characterized by the deposition of abnormally folded species of the microtubule-associated tau protein predominantly in neurons, extracellular space, and glia (Zhang et al. 2022). They are accountable for most dementias worldwide, such as Alzheimer’s disease (AD), Frontotemporal lobar degeneration (FTLD), progressive supranuclear palsy (PSP) and corticobasal degeneration (Wang and Mandelkow 2016; Götz et al. 2019; Zhang et al. 2022). Tau promotes cytoskeletal structure by catalyzing microtubule polymerization (Wang and Mandelkow 2016; Silva and Haggarty 2020; Zhu et al. 2022). Physiological tau is a highly soluble, natively unfolded protein that has little tendency to aggregate (Chang et al. 2021). Under pathogenic conditions, tau-microtubule binding dynamics and equilibrium are disturbed, resulting in tau aggregation and oligomerization to form neurofibrillary tangles (NFTs) (Wang and Mandelkow 2016). Phosphorylation serves as a physiological post-translational modification which regulates the binding of tau to microtubules (Alonso et al. 2018). Tau in NFTs is frequently hyperphosphorylated which tends to self-assemble in the cytosol and loses its affinity for microtubules. This suggests that elevated phosphorylation is a prevalent factor that strongly correlates with tau aggregation in pathological conditions, such as AD (Wang and Mandelkow 2016; Alonso et al. 2018).

In order to explore tauopathies, researchers have created a number of tau transgenic mice lines (Denk and Wade-Martins 2009). P301S mice harbor the human P301S missense mutation at exon 10 at microtubule binding domain R2 of tau protein (Yoshiyama et al. 2007). At three months of age, these animals exhibit clinical phenotypes of both PSP and FTLD, including an accumulation of NFTs in the hippocampus region, neuroinflammation, neurodegeneration, and synaptic loss (Yoshiyama et al. 2007; Creed et al. 2024). This modular progression of the disease enables the investigation of mechanisms in the hippocampus that are specifically causing synaptic dysfunction and loss at the early stages of tauopathy.

The gut microbiota (GM), which populates the human gastrointestinal system, is made up of around 100 trillion bacteria, archaea, and eukaryotes that collectively encode over 3 million genes and produce a wide range of metabolites (Valdes et al. 2018). The GM actively modulates host metabolic processes (Lynch and Pedersen 2016; Long-Smith et al. 2020; Fan and Pedersen 2021) via the bidirectional “gut-brain axis” (Martin et al. 2018; Cryan et al. 2019). Among healthy people, balanced GM composition is crucial in maintaining the integrity of intestinal barrier and suppressing the inflammation, which favorably regulates behavior and brain development via the gut -microbiota -brain axis (Buford 2017; Cattaneo et al. 2017; Sun et al. 2019). Leaky gut is a condition where intestinal barrier integrity is impaired, and permeability is increased due to resident inflammation in the gastrointestinal tracts caused by GM dysbiosis (Obrenovich 2018). After that, bacterial products such as cytokines and lipopolysaccharide (LPS) pass into the circulation through the impaired barrier, resulting in systemic inflammation (Buford 2017). Subsequently, these inflammatory mediators can reach the blood-brain barrier (BBB) and destroy it resulting in neuroinflammation and neurodegeneration (Elahy et al. 2015).

Additionally, abnormal lipid metabolism has been identified as the third pathogenic hallmark of AD, which is associated with disease progression (Luo et al. 2024). Cell membranes contain a class of metabolic lipids called sphingolipids (SPL). They comprise a bioactive subgroup which controls a variety of cellular and biological functions, including cell survival, proliferation as well as apoptosis (Hannun and Obeid 2008). Remarkably, SPL are important structural elements of gastrointestinal membranes, protecting the intestinal mucosa and controlling intestinal absorption process (Bock et al. 2007; Oertel et al. 2017). Recent research shows that GM-derived SPL can alter levels of ceramide and regulate the intestinal homeostasis **(**Johnson et al. 2020). Bacteroidota, a dominant bacterial phylum within the GM, is the only known group which can produce SPL (Olsen and Jantzen 2001). Inoculation of germ-free mice with an SPL-deficient *Bacteroides* strain altered the ceramide distribution in the cecum and exacerbated gut inflammation **(**Brown et al. 2019). According to previous studies, sphingomyelinase is activated by cytokines such as tumor necrosis factor α (TNF-α), which raise the levels of ceramides in various tissues including the colon and cell lines **(**Hannun and Linardic 1993; Colell et al. 2002; Homaidan et al. 2003). Moreover, certain bacterial toxins, such as LPS, raise ceramide levels via a toll like receptor 4 (TLR4) dependent mechanism (Fischer et al. 2007). The phospholipase enzyme known as acid sphingomyelinase (ASM) is responsible for hydrolyzing sphingomyelin into ceramide. Numerous cellular stressors, including inflammatory cytokines and pathogens can trigger ASM (Beckmann et al. 2014; Maceyka and Spiegel 2014; Zhang et al. 2024). For example, ASM inhibition has been found to reduce LPS-mediated inflammation and provide protection in mice with chemically induced colitis (Sakata et al. 2007; Xiong et al. 2019).

Amitriptyline (AMI) is one of the classic tricyclic antidepressants that inhibits ASM activity (Albouz et al. 1981; Yoshida et al. 1985) by displacing the enzyme from its membrane-bound substrate (Kölzer et al. 2004). As a result, the lysosomal enzyme degrades with a higher rate (Hurwitz et al. 1994). Due to this peculiar mechanism, these pharmacological agents have been defined as functional inhibitors of ASM (FIASMA) (Kornhuber et al. 2010). Therefore, the aim of this study was to investigate the role of AMI, as one of FIASMA drugs on colonic ASM activity, gut dysbiosis, SPL metabolism in the colon, colonic inflammation, intestinal barrier disruption and the subsequent effects on tauopathy related markers in the brain in P301S mice through the bidirectional gut-brain axis.

## Materials and Methods

### Animals

Ten male C57BL/6 mice (25–30 g) and twenty male P301S transgenic mice (20–22 g) were obtained from the Medical Experimental Research Centre (MERC), Faculty of Medicine, Mansoura University. For acclimatization, mice were monitored for a week prior to the start of experiment. Throughout the experimental period, the animals were housed in an air-conditioned room (25 ± 2 °C) and alternating 12-hour day/night cycles under standard environmental as well as nutritional conditions. All animal experiments were performed in accordance with ARRIVE guidelines (Kilkenny et al. 2010) as well as U.K. Animals Act, 1986 with ethical approval obtained from the Research Ethics Committee, Faculty of Pharmacy, Cairo University, Cairo, Egypt under the Memorandum No. PT (3260).

### Drugs and Chemicals

AMI was donated by Kahira Pharmaceuticals & Chemical Industries Company, Egypt. Methanol and chloroform that were used for preparation of the lipid extracts, were of HPLC grade and purchased from Sigma Aldrich, St. Louis, USA. Acetic acid, acetonitrile, ammonium acetate, isopropanol, and water, used for Ultra-performance liquid chromatography-mass spectrometry (UPLC-MS) analysis, were of UPLC grade and supplied by BioSolve Chimie, France. All other solvents and chemicals utilized were of the highest commercial quality and purity.

### Experimental Design

Wild type C57BL/6 mice represented the control model, while P301S transgenic mice were randomly assigned into two groups which represent tauopathy model. Thereafter, the experimental design consisted of the following groups: (i) Control C57BL/6 mice group: received no treatment. (ii) Tauopathy P301S transgenic mice group: received no treatment. (iii) AMI-treated P301S transgenic mice group: received AMI dissolved in drinking water at dose of (180 mg/L) for 35 days, as previously described by a previous study (Gulbins et al. 2013).

After a 35-day treatment period, fecal samples were collected, and behavioral testing was performed on each animal. After that, thiopental (EIPICO, Egypt, 100 mg/kg, ip.) was used to deeply anesthetize the mice, and blood was obtained by cardiac puncture followed by centrifugation to obtain the serum. Subsequently, the mice were transcardially perfused for 2 min using 50 ml of 0.1 M phosphate buffered saline (PBS). Then, 150 ml of 4% paraformaldehyde was perfused into the half of each group that had been prepared to undergo histopathological analysis to fix the brain and colon. Following the decapitation of the mice, the brains and the colonic tissues were quickly and carefully excised. The aforementioned half of each group’s brains and colons were fixed in 4% paraformaldehyde and prepared to make paraffin blocks which were then utilized for histopathological evaluation. The second half of the mice brains of each group were bisected into two hemispheres on ice for separation of hippocampus, and their colonic tissues were also removed, rapidly frozen, and kept at −80 °C for further lipidomic and biochemical assessment.

### Behavioural Tests

After a 35-day treatment period, neurobehavioral activity of mice was evaluated using ANY-box^®^ (Stoelting Company, USA) apparatus for behavioral testing, Y maze apparatus, and morris water maze (MWM) apparatus in a sound-isolated room. Behavioral performance was analyzed using ANY-maze™ software. Four neurobehavioral tests were performed as follows.

#### Morris Water Maze (MWM)

The MWM was used to investigate hippocampus-dependent spatial learning and memory impairments. the test was carried out in a cylindrical basin (110 cm in diameter), with a white painted walls, filled with water, as originally described by Richard Morris with minor modifications (Morris 1984; Mohamed et al. 2024). Also, a white platform was used throughout the training days and kept in a fixed position. MWM experiment consists of six days: Day 0 assigned as pre-training phase, Days 1–4 assigned as acquisition phase, and Day 5 assigned as probe trial. Regarding the pre-training phase, mice were allowed to freely swim in the water for 60 s with a visible platform positioned above water level. During acquisition phase, mice were carefully placed facing the arena wall. They were then allowed to swim to the platform, which was below the water level and allowed to stay on it for 10 s. Every day, each mouse receives four 120-second trials, one from each quadrant. The latency period was recorded for all days as the time taken to reach the platform by each mouse from each quadrant. During the given 120 s in the acquisition phase, mice which failed to reach the platform were gently guided to the platform (without being lifted out of the water), kept for 20 s on the platform and recorded with a latency period of 120 s. The escape latency, which was used as an indicator for spatial learning, was calculated as the mean of the total time spent until reaching the platform across all trials for each day of the acquisition phase. The water was made opaque by adding starch and remained unchanged during the trial days, except for clearing away any floating fecal pellets. Regarding the probe trial, the platform was removed then, each mouse was carefully placed in the water in the quadrant opposite to the platform quadrant in acquisition phase, facing the pool wall, and given 60 s to freely swim. After each trial, the mice were dried off and placed under a heat lamp to keep their bodies warm. The temperature of the water was kept at 24 °C. ANY-maze™ software was used to analyze recorded videos of probe trial.

#### Y Maze

The Y maze test, which recorded the spontaneous alternation of mice, was used to evaluate of the short-term spatial memory in mice. Briefly, mice were positioned at the end of one arm and then allowed to freely explore the wooden Y maze apparatus for 8 min with dimension of 40 cm in length × 10 cm in height (Ma et al. 2007). The arms of the apparatus was labeled as A, B, and C and set at 120 degrees. The spontaneous alternation behavior as well as number of entries into each arm were evaluated to assess motor activity alongside mice’s tendency to alternate between the different arms, respectively. Certain arm transitions sequences (ABC, CBA, BCA, ACB, CAB or BAC rather than CBC, CAC, ABA, ACA, BCB or BAB) were recorded as a spontaneous alternation which indicates short-term spatial memory. 10% ethanol was used to clean the maze’s arms in between sessions. The following formula was used for calculating the percentage of spontaneous alternation: [(Number of spontaneous alternations of each mouse)/(Total number of entries into Y maze arms − 2)] × 100 (Kouémou et al. 2017).

#### Novel Object Recognition (NOR)

The NOR test was performed as previously described in (Lueptow 2017) with slight modifications. An acrylic clear plastic box with transparent walls of dimensions (40 × 40 × 35) cm (width, length, height) was utilized to carry out the NOR test. Two discriminating objects (novel object: cone shaped box and familiar object: rectangle box) of identical height and material were utilized to carry out NOR test. NOR test consists of three phases: (Day 1) habituation phase, (Day 2) familiarization phase, and (Day 3) testing phase. For five minutes during the habituation period, each mouse was allowed to freely explore the box without any objects. After 24 h, the familiarization phase began, and the mouse was given five minutes to freely explore the box, which contained two similar familiar objects. After 24 h, the test phase was undertaken. During the test phase, the mouse was given five minutes to freely explore the box, which now contained two discriminating objects (a novel object as well as a familiar object), for 5 min. Videos of test phase were recorded and analyzed using ANY-maze™ software. During the test phase, the degree of a mouse to distinguish between familiar object and novel object was calculated as the Discrimination Index (DI): DI = N/N + F where N is the mouse exploration time of novel object, and F is the mouse exploration time of familiar object during the test phase. The apparatus and objects were cleaned to remove odor cues before and between use.

#### Parallel Rod Floor Test

The Parallel rod floor apparatus is made up of a transparent plastic acrylic box of dimensions 20 × 20 × 30 cm (width, length, height) and several parallel rods made of stainless-steel located at a base plate made of stainless-steel which served as the chamber’s floor. The neurobehavioral motor activity was evaluated by assessing the number of Foot slips (number of errors) that were counted by a sensor beneath the floor. percentage of errors per meter was calculated by dividing the number of foot slips of each mouse by the total distance travelled by the mouse in meters (Kamens and Crabbe 2007).

### Histopathological and Histochemical Examination

#### Hematoxylin and Eosin (H&E)

Colonic and brain tissues were washed, preserved then fixed in 10% neutral buffered formalin for 72 h. Then, samples were cut, treated graded ethanol series, cleared with Xylene, and embedded into Paraplast embedding media. A rotatory microtome was used for cutting 5µn thick tissue sections for the demonstration of full colonic wall as well as hippocampal regions. H&E was used as a general staining procedure for morphological examination of slices of brain and colonic tissue. Additionally, colonic tissue sections only were stained with Alcian blue (pH 2.5) to quantify goblet cells and reactive acidic mucins. Afterwards, all slides were blindly examined under a microscope by skilled histologist. All standard techniques for staining and tissue fixation complies with Culling (Culling 2013).

#### Immunohistochemistry

Colonic tissue sections that were five microns thick and embedded in paraffin were prepared for immunohistochemical analysis in accordance with the protocol of manufacturer. The retrieved colonic sections were deparaffinized, exposed to 0.3% H_2_O_2_ for 20 min, followed by incubation with antibody against Zonula Occludens-1 (ZO-1) (Abcam - Cat. No. EPR19945-224–1:1000) or antibody against Claudin 1 (Thermofisher - Cat. No. 37–4900–1:100) at 4 °C overnight. Then, colonic sections were rinsed with PBS, treated for twenty minutes with the HRP secondary antibody Envision kit (DAKO), rinsed, and incubated for 15 min with diaminobenzidine, rinsed with PBS, counterstained with hematoxylin, dehydrated, and cleared before being covered and examined using a microscope.

#### Histological Analysis

To determine the number and diameter of goblet cells stained by alcian blue in colonic tissue sections as well as mucosal reactive mucin, at least six randomly selected non-overlapping fields from each group were scanned in accordance with (Khedr et al. 2023). Also the percentage area of immunoexpression levels of Claudin 1 as well as ZO-1 were assessed in immunohistochemically stained sections. The Leica Application module for histological analysis, which is connected to the Full HD microscopic imaging system (Leica Microsystems GmbH, Germany), was used for all light microscopic examinations and data processing.

### Preparation of Tissue Homogenate

After sacrifice, colonic and hippocampal tissues were washed thoroughly and rinsed with ice. A polytron homogenizer was used at 40 °C to prepare 10% of tissue homogenate in 0.05 M PBS (pH 7). To get rid of the cell debris, intact cells, nuclei, erythrocytes, and mitochondria, the homogenate was centrifuged for 20 min at 10,000 rpm.

### Enzyme-Linked Immunosorbent Assay (ELISA)

Serum, along with the supernatant obtained after centrifugation of the tissue homogenate, was utilized to estimate the protein levels in the serum, hippocampal tissue besides distal colon tissue in accordance with guidelines provided by the manufacturer. All ELISA kits: Nuclear Factor Kappa B (NF-KB) (MyBioSource, San Diego, CA, USA, Cat. No. MBS043224), Myeloid Differentiation Factor 88 (MyD88) (MyBioSource, San Diego, CA, USA, Cat. No. MBS2533515), TNF-α (MyBioSource, San Diego, CA, USA, Cat. No. MBS825075) and interleukin-22 (IL-22) (MyBioSource, San Diego, CA, USA, Cat. No. MBS8291422) were measured in the distal colon by mouse ELISA kit. Moreover, brain-derived neurotrophic factor (BDNF) was measured by (MyBioSource, San Diego, CA, USA, Cat. No. MBS355435) mouse kit in hippocampus. LPS was measured in serum by using (MyBioSource, San Diego, CA, USA, Cat. No. MBS261904) mouse ELISA kit by using an ELISA plate reader.

### Colorimetric Assay

ASM colorimetric assay kit (Abcam, Cambridge, UK, Cat. No. ab252889) was used for measuring ASM activity at (OD 570 nm) in accordance with manufacturer instructions in the supernatant remained after centrifugation of the distal colon homogenate.

### Western Blot Analysis

Hippocampus protein was extracted using ice-cold RIBA lysis buffer PL005 (Bio Basic Inc., Markham, Ontario, L3R 8T4 Canada), then centrifugation was performed at 4 °C at 16,000xg for 30 min. Then, The Bradford Protein Assay Kit (SK3041) (Bio Basic Inc., Markham, Ontario, L3R 8T4 Canada) was used to measure the protein concentration in the supernatant in accordance with the instructions provided by the manufacturer. Using an equal volume of 2x Laemmli sample buffer, each sample was loaded with 20 µg of protein at pH = 6.8. The samples then were boiled for 5 min at 95 °C to denaturize the protein, sonicated for 30 s, centrifuged for 10 min at 10,000 g, separated based on molecular weight using sodium dodecyl sulfate–polyacrylamide gel electrophoresis, relocated into PVDF membranes, blocked by tris-buffered saline with Tween 20 (TBST) along with 3% bovine serum albumin at room temperature for 1 h and then incubated with the specific primary antibody against either phosphorylated tau protein (P-tau) (Thermo Fisher Scientific, Cat. No. MN1010) or protein phosphatase 2 A (PP2A) (Thermo Fisher Scientific, Cat. No. MA5-18060) after being diluted with TBST overnight at 4 °C in accordance to manufacturer instructions. Then, the blot was washed with TBST for 5 min 3–5 times and incubated for one hour with HRP-conjugated secondary antibody (Goat anti-rabbit IgG, Novus Biologicals, USA, 1:5000 dilution) before the blot was exposed to the chemiluminescent substrate (Clarity™ Western ECL substrate Bio-Rad cat. No 170–5060). A CCD camera-based imager was utilized to capture chemiluminescent signals. The target proteins’ band intensities were compared to the control sample beta actin using image analysis software and protein normalization on the ChemiDoc MP Imaging System (Bio-Rad, California, USA).

### Fecal Sample Collection

The mice were placed in a clean plastic box that had been sterilized with 70% alcohol no more than one hour prior to fecal sample collection. Fecal pellets were collected by using sterile forceps and aseptically placed inside sterilized 2 ml Eppendorf tubes. After a 35-day treatment period, pooled fecal samples were collected from each group in 2 ml Eppendorf tubes and then stored at −80 °C until further analysis.

### DNA Extraction and Quantification

DNA was extracted from 9 fecal samples, representing the three experimental groups, using QIAamp Fast DNA Stool Mini extraction kit (Qiagen, Hilden, Germany). Then, the extracted DNA was instantly stored at −20 °C. Nucleic acid concentrations were initially measured using a Nanodrop spectrophotometer and subsequently validated via the Qubit^®^ fluorometric system (Life Technologies, Carlsbad, CA), using Qubit dsDNA Hs assay kit (Life Technologies corporation, Oregon, USA). Furthermore, nucleic acid purity (260/280 and 260/230 absorbance ratios) was determined.

### 16 S rRNA Amplicon Sequencing

Concentrated DNA, with high purity, extracted from each of the nine pooled fecal samples across different groups, was sequenced at The Center for Genome and Microbiome Research in Cairo, Egypt, using an iSeq™100 platform (Illumina, San Diego, USA). An Illumina amplicon library, targeting the V3-V4 hypervariable regions of the bacterial 16 S rRNA gene, was prepared following a 2 × 150 bp paired-end protocol. Amplification of the targeted hypervariable regions was performed according to the manufacturer’s 16 S rRNA metagenomic sequencing library preparation guidelines, via PCR with degenerate primers; 515 F (5′- TCGTCGGCAGCGTCAGATGTGTATAAGAGACAGGTGYCAGCMGCCGCGGTA- 3′) and 806R (5′- GTCTCGTGGGCTCGGAGATGTGTATAAGAGACAGGGACTACHVGGGTWTCTAAT-3′).

Conditions for PCR cycling were as follows: 95 °C for 3 min followed by 25 cycles of 95 °C for 30 s, 52 °C for 30 s, 72 °C for 30 s then final extension at 72 °C for 10 min. Following purification by AmpureXP beads (Beckman Coulter, Brea, CA, USA), the PCR products were eluted in 10 mM Tris elution buffer. Dual indices and Nextera XT Index primers were then attached by PCR (95 °C for 3 min followed by 8 cycles for 30 s at 95 °C, 55 °C for 30 s, 72 °C for 30 s then final extension at 72 °C for 10 min. A Qubit^®^ fluorometer (Life Technologies, Carlsbad, CA, USA) was used to quantify the libraries, whose integrity was further assessed by agarose gel electrophoresis. Following validation, the libraries were finally pooled, in equimolar ratios, into a single amplicon library and loaded in an Illumina iSeqTM 100 i1 Cartridge kit (Illumina, San Diego, CA, USA).

### Microbiome Analysis

Following automated base calling by the built-in BaseSpace software (Illumina), the raw sequence reads retrieved from the iSeq instrument were processed by MOTHUR software v.1.36.1 (Schloss et al. 2009). The sequence analysis followed the MiSeq standard operating methodology (URL: https://mothur.org/wiki/miseq_sop, accessed on 1 February 2025) (Caporaso et al. 2012; Kozich et al. 2013). Sequences having at least one ambiguous base or more than eight homopolymers, short sequences, and sequences with unknown base pairs (sequences with N’s), were removed and excluded from downstream analysis.

MOTHUR taxonomic assignment was generated, following the Wang approach (Wang et al. 2007), against SILVA bacterial database version 138 (Quast et al. 2013). After clustering the sequences into operational taxonomic units (OTUs) based on a 97% cutoff for sequence similarity, chimeric sequences were detected and removed using UCHIME (Edgar et al. 2011).

MicrobiomeAnalyst 2.0, a web-based platform for visual as well as statistical analysis of microbiome data, was used to analyze OTUs abundance along with consensus taxonomy output files (Lu et al. 2023). Sequence reads were first subjected to denoising using a minimum prevalence threshold of 20% and a count cutoff of 4. Normalization and rarefaction of the data to the minimum library size were then applied, followed by total sum scaling. Low-variance features were then filtered using a standard deviation (SD) cutoff of 5%. Alpha diversity metrics- Chao1 richness, ACE, Fisher, Simpson, and Shannon indices- were calculated and evaluated for statistical significance via the Kruskal-Wallis test.

Beta diversity was assessed using the permutational multivariate analysis of variance (PERMANOVA) statistical method, which relies on Bray–Curtis dissimilarity, and visualized through principal coordinate plots using default cutoffs. Taxa with significant differences were identified using linear discriminant analysis effect size (LEfSe). The false discovery rate (FDR) method was used to adjust *P* values (Segata et al. 2011).

### Preparation of Lipid Extracts

The lipid extracts were prepared in accordance to previously described method (Schiffmann et al. 2009) with some minor modifications. Briefly, samples (150 mg) of distal colon derived from mice from each experimental group (P301S model, AMI-treated, and control groups) were individually homogenized with PBS in presence of ice. A sample (50 µL) of each tissue homogenate was diluted with 450 µL of prechilled PBS then added to a 7:1 mixture of chloroform: methanol (3.5 mL) to extract lipids. The tissue homogenate-organic layer mixture was vortexed for 1 min at room temperature, followed by centrifigation for 15 min at 5000 r.p.m. at room temperature. The organic phase was separated and the extraction step repeated. The combined organic phase was vacuum-dried at 50 °C using a Rotavapor^®^ R-100 rotatory evaporator (Büchi, Switzerland).

### UPLC-MS Analysis of the Lipid Extract

The UPLC-MS analysis of the tissue lipids was performed in accordance with a previous study (Salem et al. 2020) with some minor modifications. The UPLC analysis was performed using UPLC system attached to an Exactive mass spectrometer (Thermo-Fisher, http://www.thermofisher.com). Chromatographic separation was done using a C8 reversed phase column (100 mm L × 2.1 mm i.d. × 1.7 μm particles; Waters, Massachusetts, USA). The mobile phases composed of water containing 0.1% acetic acid and 1% 1 M ammonium acetate, as Buffer A, and acetonitrile mixture: isopropanol in 7:3 ratio that contains 0.1% acetic acid and 1% 1 M ammonium acetate, as Buffer B. The dried lipid extracts (6 mg each) were individually suspended in a mixture of acetonitrile: isopropanol in 7:3 ratio (500 µL), then 2 µL of each sample were loaded per injection. The flow rate was set to 400 µL/minutes and the applied gradient was: 45% of buffer A for 1 min, linear gradient from 45% to 35% of buffer A for 3 min, linear gradient from 25% to 11% of buffer A for 8 min, linear gradient from 11% to 1% of buffer A for 3 min. After washing the column for 3 min with 1% buffer A, the mobile phase was set back to 45% A and the column was re-equilibrated for 4 min (the total run time was 22 min). The spectra, which covered a mass range of 100–1500 m/z, were alternately recorded in full-scan and all-ion fragmentation-scan modes. The loading time was limited to 100 ms, and the resolution was set to 10,000 with 10 scans per second. With a sheath gas flow value of 60 as well as an auxiliary gas flow of 35 (values are in arbitrary units), the capillary voltage was adjusted at 3 kV. The drying gas and capillary temperature in the heated electrospray source were adjusted at 350 and 150 °C, respectively. The skimmer voltage was adjusted at 25 V, while the tube lens was adjusted at a value of 130 V. The spectra were recorded from 1 to 17 min of the UPLC gradients.

### Statistical Analysis

Except for GM analysis, all statistical analysis was performed using GraphPad Prism 9.5.1. All data are presented as a mean ± SD. Data were tested for normality using the Shapiro–Wilks test. parametric data was analyzed using One-way analysis of variance (ANOVA) followed by post hoc Tukey’s multiple comparison analysis, while nonparametric data was analyzed using Kruskal Wallis tests followed by Dunn’s multiple comparison test. A Probability value *(p*) below 0.05 was considered significant where *p ≤ 0.05, **p ≤ 0.005, ***p ≤ 0.001 and ****p ≤ 0.001.

## Results

### Effects of AMI Treatment in P301S Mice on Long-Term Spatial Memory

**Fig. 1:**
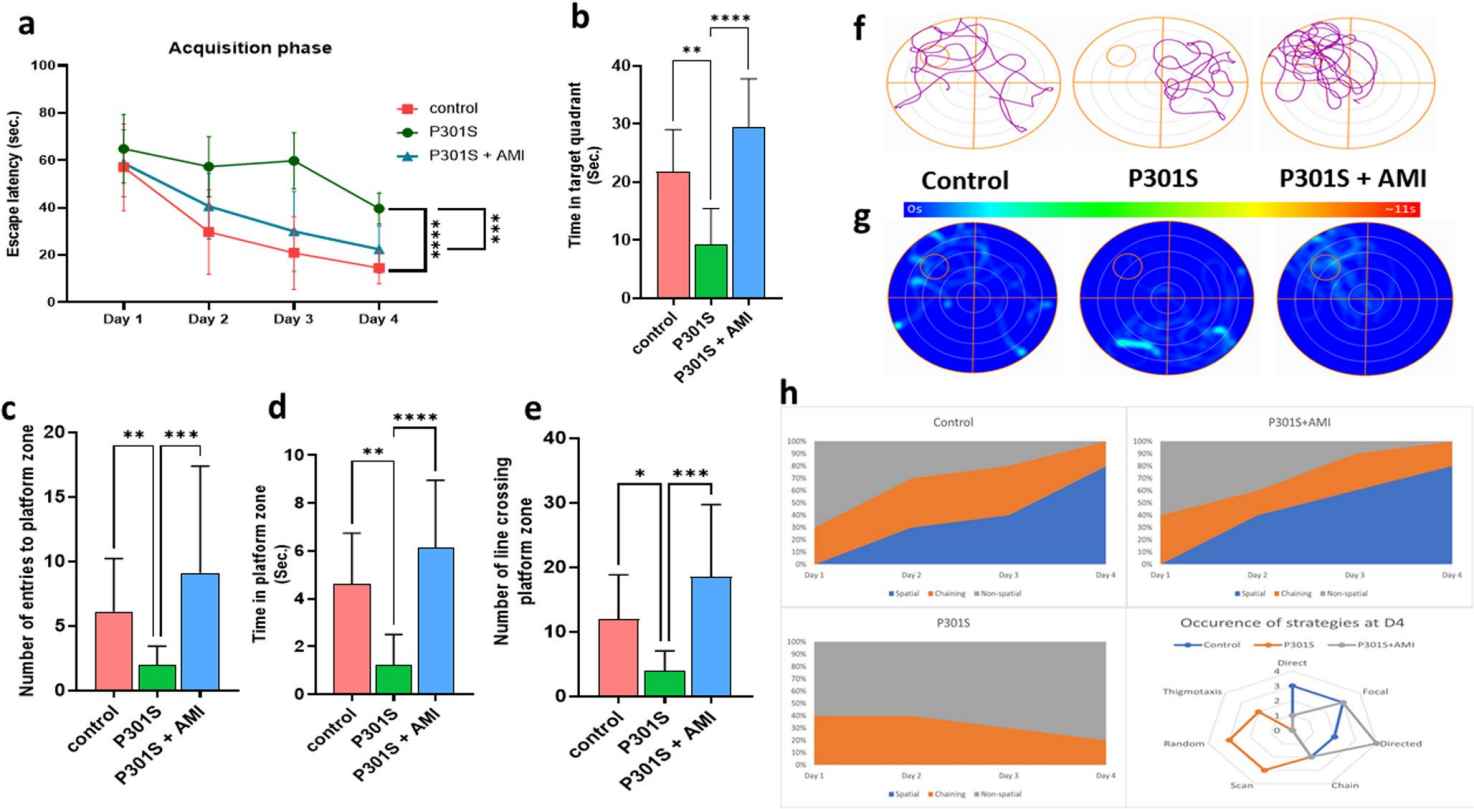
Effect of AMI on Spatial learning and long-term memory in P301S mice in the acquisition and probe phases of the MWM test. (**a**) Escape latency in the four days of the acquisition phase. (**b**) Time in target quadrant (**c**) Number of entries to the platform zone. (**d**) Time spent in platform zone. (**e**) Number of lines crossing platform zone. (**f**) Track plots indicate the movement of different mice groups in MWM (**g**) Heat maps indicate the movement of different mice groups in MWM (**h**) Radar chart representing search strategies of the different groups at acquisition day 4. All data are presented as a mean ± SD. (*n* = 10). One-way analysis of variance (ANOVA) was used to determine statistical significance, followed by Tukey’s multiple comparison analysis except for the number of entries to the platform zone and number of lines crossing platform zone which were tested using Kruskal–Wallis test to determine statistical significance, followed by Dunn’s multiple comparison test. A p-value below 0.05 was considered significant where *p ≤ 0.05, **p ≤ 0.005, ***p ≤ 0.001, ****p ≤ 0.001

Long-term spatial memory was assessed using MWM. The MWM results demonstrated that AMI treated P301S mice’s escape latency was significantly and gradually decreased with increasing training days compared to tauopathy model. However, the escape latency of P301S mice fluctuated across training days and significantly higher than control group (Fig. 1a). The longer the time spent in target quadrant and platform zone and the larger the number of line crossings of platform zone and number of entries to platform zone, the better the long-term spatial memory and learning of mice. The results of the long-term spatial exploration experiment showed that P301S mice have significant decreased time in target quadrant, time in platform zone, number of entries to platform zone and number of lines crossing the platform zone by 60% (*P* = 0.0021, F _(2,27)_ = 19.11),75% (*P* = 0.0047, F _(2,27)_ = 13.54), 60% (*P* = 0.0096), 39% (*P* = 0.0391) respectively comparing to control group. However, AMI treatment in P301S mice showed significant increase in time in target quadrant, time in platform zone, number of entries to platform zone and number of lines crossing the platform zone by 4.7 folds (*P* < 0.0001, F _(2,27)_ = 19.11), 10.8 folds (*P* < 0.0001, F _(2,27)_ = 13.54), 4.5 folds (*P* = 0.0006), 5.8 folds (*P* = 0.0007) respectively when compared to tauopathy model (Fig. 1b-e**)**. These results were obviously demonstrated in corresponding track plots (Fig. 1f) and heat maps (Fig. 1g) of different groups.

### Effects of AMI Treatment in P301S Mice on Short-Term Spatial Memory

**Fig. 2:**
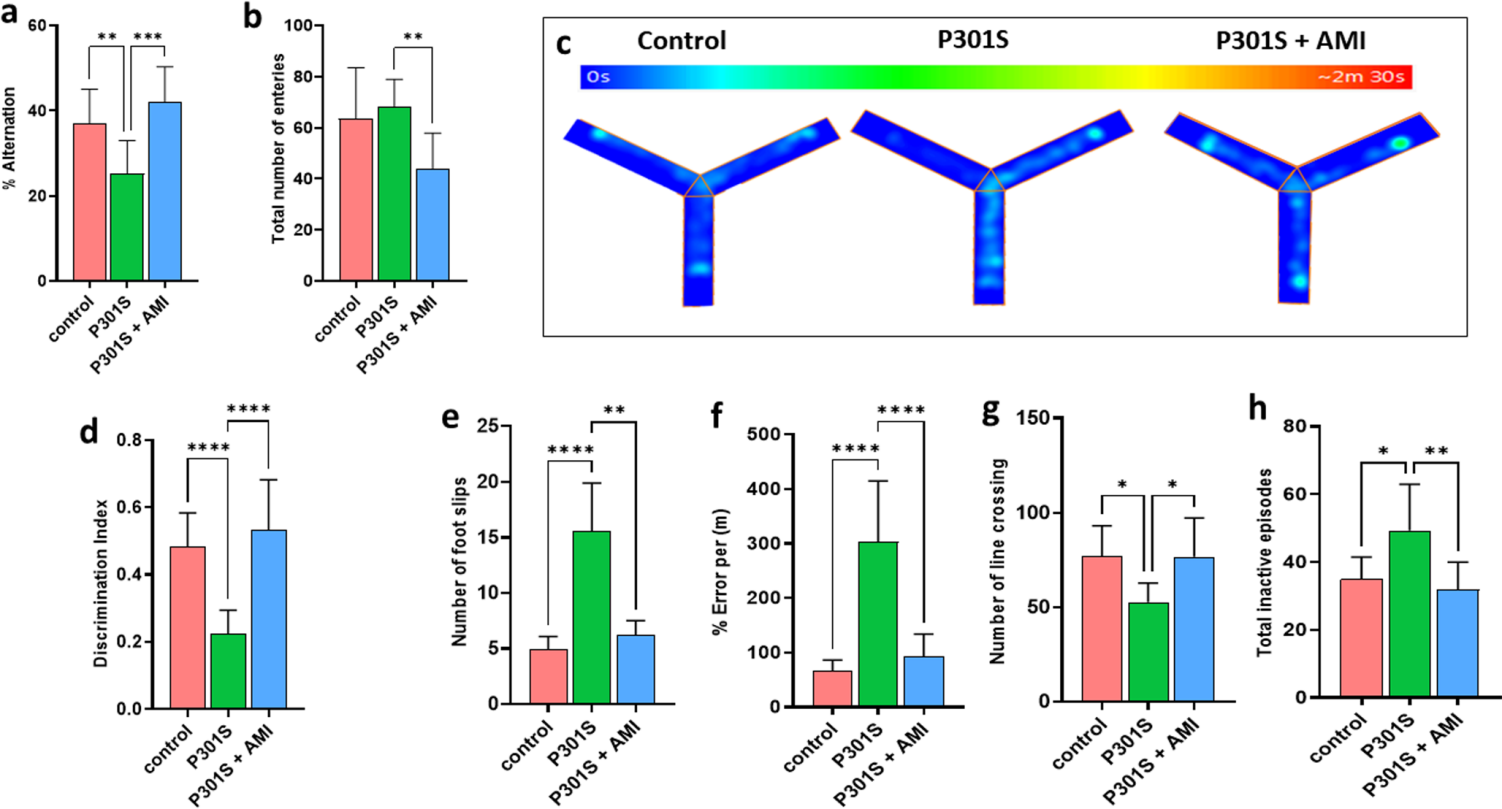
Effect of AMI on short-term spatial memory in Y-maze, recognition memory in NOR test and motor incoordination in parallel rod floor test in P301S mice. (**a**) Spontaneous Alternation percentage in Y-maze. (**b**) Total number of arm entries in Y-maze. (**c**) Heat maps indicate the movement of different mice groups in Y-maze. (**d**) Discrimination index of different mice groups in NOR test (**e**) Number of foot slips in parallel rod floor. (**f**) Percentage of error per meter in parallel rod floor (**g**) Number of lines crossings in parallel rod floor. (**h**) Total inactive episodes in parallel rod floor. All data are presented as a mean ± SD. (*n* = 10). Kruskal–Wallis was used to determine statistical significance, followed by Dunn’s multiple comparison test except for Spontaneous Alternation percentage in Y-maze, Discrimination index of different mice groups in NOR test and Percentage of error per meter in parallel rod floor which were tested One-way analysis of variance (ANOVA) was used to determine statistical significance, followed by Tukey’s multiple comparison analysis A p-value below 0.05 was considered significant where *p ≤ 0.05, **p ≤ 0.005, ***p ≤ 0.001, ****p ≤ 0.001

Regarding the Y maze test, there was no significant difference in the total number of entries to Y maze arms between P301S mice and control group. But AMI treated P301S mice showed significant decrease in total number of entries to Y maze arms by33% (*P* = 0.0043) when compared to tauopathy model. Spontaneous alternation was significantly lowered in P301S mice by 28% (*P* = 0.0075, F _(2,27)_ = 11.11) when comparing to control group and increased significantly by 1.8 folds (*P* = 0.0003, F _(2,27)_ = 11.11) in the AMI treated P301S mice comparing to tauopathy model (Fig. 2a&b). Higher alternation rate reflects the better spatial exploration ability of mice.

### Effects of AMI Treatment in P301S Mice on Recognition Memory

Recognition memory was assessed using NOR test. DI was significantly decreased in tauopathy model by 51% (*P* < 0.0001, F _(2,27)_ = 21.82) comparing to control group. However, AMI treated P301S mice showed significant increase in DI by 2.5 folds (*P* < 0.0001, F _(2,27)_ = 21.82) when compared to tauopathy model (Fig. 2d).

### Effects of AMI Treatment in P301S Mice on Locomotor Activity Impairment and Motor Incoordination

Motor incoordination and impairment of locomotor activity in P301S mice was assessed using parallel rod floor test. Evidently, Motor incoordination and impairment of locomotor activity in tauopathy model was clearly noticed in several parameters such the increase in number of foot slips by 3.5 folds (*P* < 0.0001), percentage of errors per meter by 5 folds (*P* < 0.0001, F _(2,27)_ = 35.23), total inactive episodes by 1.5 folds (*P* = 0.0439) and decrease in number of lines crossing by 30% (*P* = 0.0108) comparing with control group. AMI treatment in P301S mice demonstrated significant improvement in motor coordination and locomotion activity by significant decrease in number of foot slips by 57% (*P* = 0.0048), percentage of errors per meter by 64% (*P* < 0.0001, F _(2,27)_ = 35.23), total inactive episodes by 24% (*P* = 0.0068) and significant increase in number of lines crossing by 1.5 folds (*P* = 0.0189) when compared to tauopathy model (Fig. 2e-h).

### Effect of AMI Treatment in P301S Mice on Acid Sphingomyelinase Activity

**Fig. 3:**
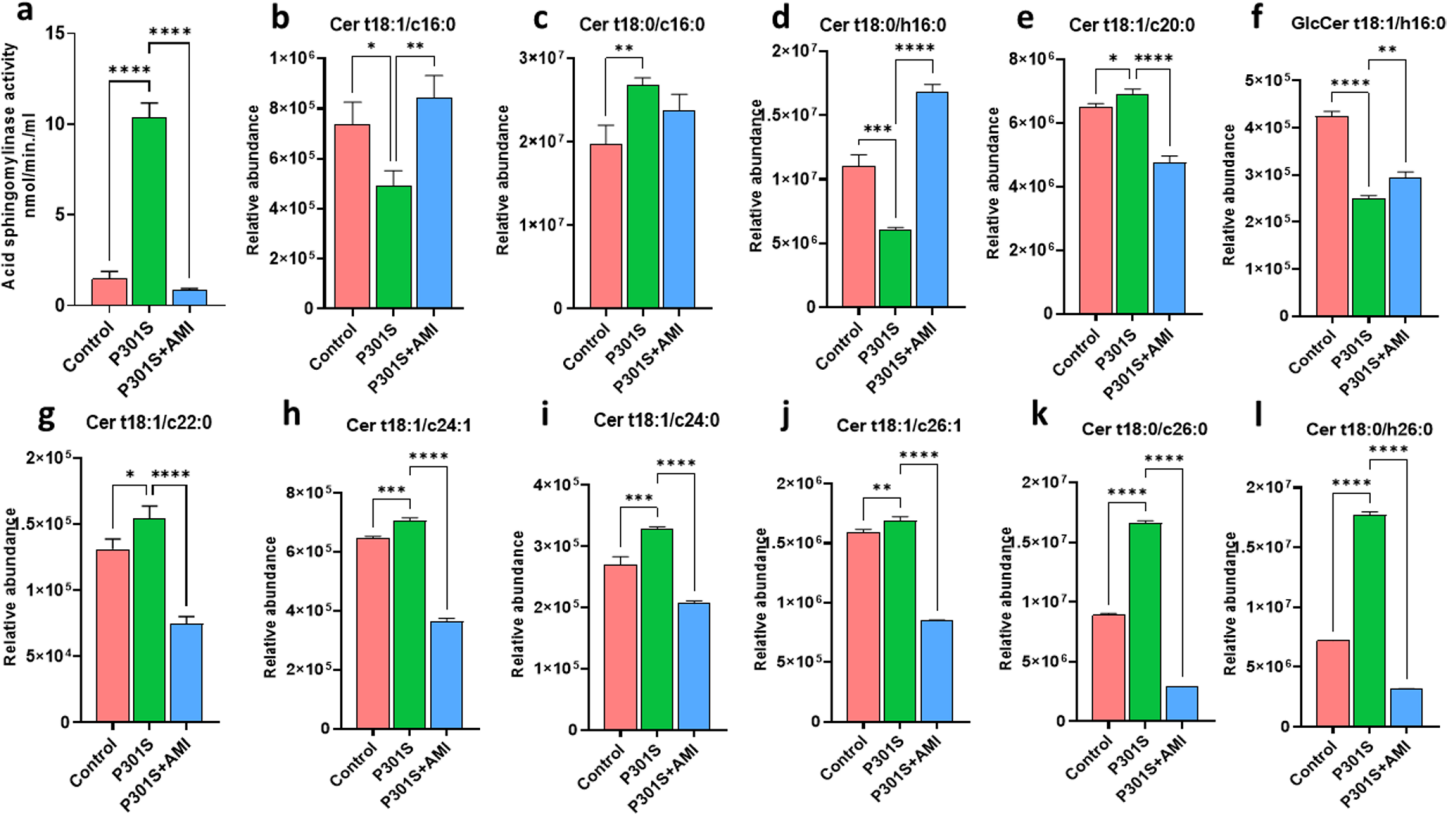
Effect of AMI treatment on ASM activity and ceramide levels on distal colon of P301S mice. (**a**) ASM activity in distal colon (*n* = 6). (**b**-**l**) Relative abundance of the identified ceramides by UPLC-MS in distal colon in the investigated groups in positive ionization mode. All data are presented as a mean ± SD. One-way analysis of variance (ANOVA) was used to determine statistical significance, followed by Tukey's multiple comparison analysis. A p-value below 0.05 was considered significant where *p≤ 0.05, **p ≤ 0.005, ***p ≤ 0.001, ****p ≤ 0.001

In order to confirm the rationale of this study based on selection of AMI as one of FIASMA drugs. ASM activity was assessed in distal colon in different groups. As shown in (Fig. 3a) P301S mice showed significant upregulation of ASM activity in distal colon by 7.5 folds (*P* < 0.0001, F _(2,15)_ = 594.4) comparing with control group. Remarkably, AMI treated P301S mice showed significant decrease in ASM activity by 92% (*P* < 0.0001, F _(2,15)_ = 594.4) when compared to tauopathy model confirming the AMI functional inhibitory activity against ASM.

### Effects of AMI Treatment in P301S Mice on Lipidomic Analysis of Distal Colon

The lipid profiles of mice distal colon tissue homogenate prepared from P301S mice group, AMI-treated P301S mice and control group were assessed through UPLC-MS. The MS/MS fragmentation patterns of the detected tissue lipids were matched with library database for their identification. Eleven ceramides were identified (Table 1). The relative abundance of endogenous ceramides in distal colon tissue homogenates were compared among the experimental groups. The results showed that eight of the identified ceramides were present in the significant highest abundance in the P301S mice model of tauopathy, as shown in (Fig. 3b-l) confirming the involvement of ceramides in development of tauopathy. Moreover, seven of these ceramides showed significantly lower abundance in AMI -treated P301S mice when compared to the tauopathy model suggesting the potent inhibitory activity of AMI against ASM enzyme.

**Table 1:** Identified ceramides in distal colon tissue homogenate of different group

No.	Rt (minutes)	(M + H)^+^	Identification
1	9.28	554.5122	Cer t18:1/c16:0
2	9.03	556.5278	Cer t18:0/c16:0
3	9.83	572.5227	Cer t18:0/h16:0
4	9.32	610.573	Cer t18:1/c20:0
5	7.88	732.5592	GlcCer t18:1/h16:0
6	10.42	638.6054	Cer t18:1/c22:0
7	10.69	664.6213	Cer t18:1/c24:1
8	11.7	666.6369	Cer t18:1/c24:0
9	8.23	692.6602	Cer t18:1/c26:1
10	12.44	696.682	Cer t18:0/c26:0
11	13.8	712.6786	Cer t18:0/h26:0

### Effects of AMI Treatment in P301S Mice on the GM Profiling by 16 S rRNA Amplicon Sequencing

To investigate any potential GM dysbiosis in the tauopathy model as well as any potential protective effect exerted by AMI, we sequenced the DNA extracted from nine pooled fecal samples, collected after 35 days treatment period from the three experimental groups. The reads of raw sequences were subjected to quality filtering and pre-processing before being analyzed using MOTHUR software against the SILVA 16 S rRNA database (version 138). Sequence reads that were classified and filtered by MOTHUR analysis, were subsequently assigned to 715 non-redundant taxonomic units of various taxonomic levels (including 413 genera, 158 families, 86 orders, 39 classes and 16 phyla). The assigned taxa were then compared across samples and sample groups at different taxonomic levels. The MicrobiomeAnalyst 2.0 was then used to re-filter, statistically analyze, and visualize all of the data (Lu et al. 2023), as follows. The analyzed reads were normalized across all samples as a fraction of total reads and then rarefied to the minimum library size (1,1821). Additionally, a low-variance filter was applied, as detailed in the Methods section, which removed 6,527 low-abundance features and 64 low-variance features.

At the phylum level, MOTHUR initially identified 16 phyla, 10 of which were retained after further analysis by MicrobiomeAnalyst. Among these, four phyla were the most abundant across all samples: Bacteroidota (with mean relative abundance = 61.57% of filtered classified reads), followed by Firmicutes (with mean relative abundance = 30.97% of filtered classified reads), Desulfobacterota (with mean relative abundance = 3.112 of filtered classified reads), and Campylobacterota (with mean relative abundance = 1.8% of filtered ones). Six other phyla were detected at low levels (Fig. supplementary 1). At the genus level, 72 genera were identified, 10 of which were the predominant genera (Fig. supplementary 2), while 62 other genera were detected with low proportions.

#### Changes in Fecal Microbiota Diversity Across Experimental Groups

The fecal microbiota alpha diversity of mice was assessed using various indices, at the phylum and the genus levels. Although no significant difference was observed between the three treatment groups at the phylum level across five alpha diversity indices (Fig. supplementary 3a), three indices—Shannon, Simpson, and Fisher—showed significant differences at the genus level (Fig. supplementary 3b), with the control group exhibiting the highest diversity.

**Fig. 4:**
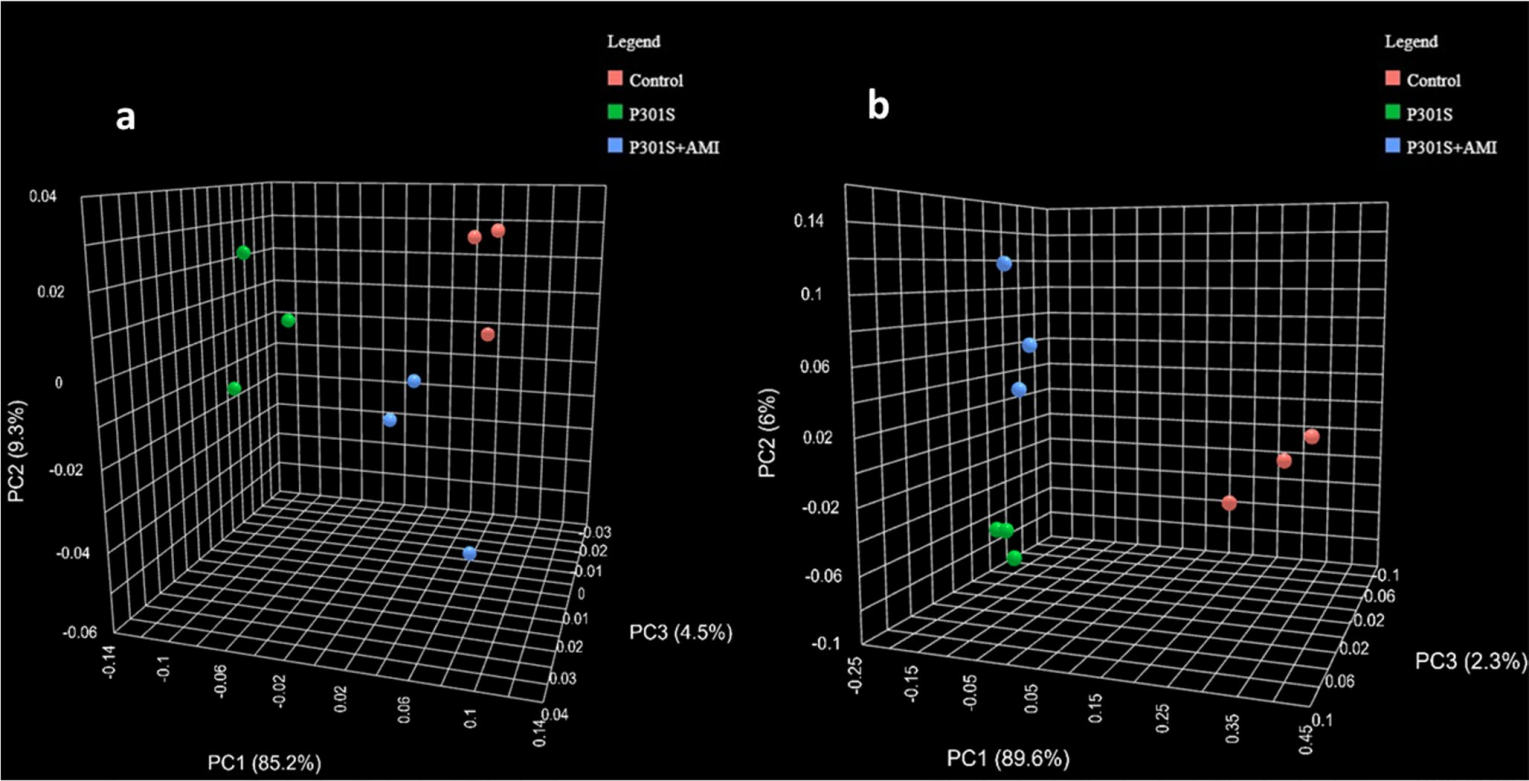
Beta-diversity, visualized by PCA of Bray–Curtis distances between different samples. (**a**) Comparison between Control, P301S, and P301S + AMI groups at the phylum level: samples are separated into three distinct clusters—PERMANOVA F-value = 13.264, R-squared = 0.8155, p value = 0.009. (**b**) Comparison between Control, P301S, and P301S + AMI groups the genus level: the samples are separated into three distinct clusters—PERMANOVA F-value = 53.75, R-squared = 0.947, p value = 0.009

The beta diversity, assessed through principal component analysis (PCA) of Bray–Curtis distances of all sequence variants, indicated clear segregation between different groups at both the phylum and genus levels (Fig. 4a and b, respectively).

#### Changes in Fecal Microbiome Profiles Across Experimental Groups

**Fig. 5:**
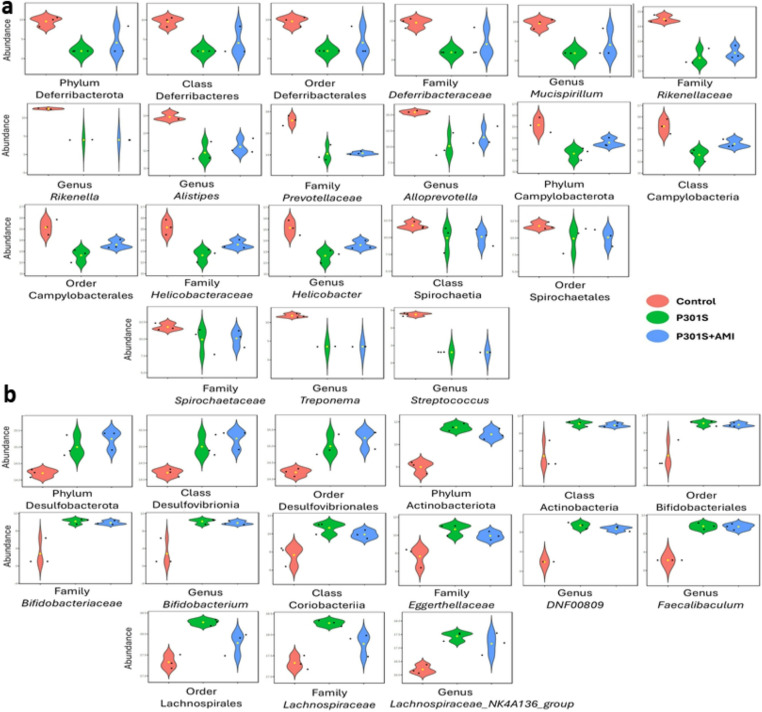
Taxa with significant differences in relative abundance among the three tested groups. Violin plots describe differential relative abundance of thirty-five significant taxa in different samples; (**a**) abundant taxa in the control group, (**b**) scarce taxa in the control group. The significance was identified by LEfSe analysis, with *p* value cutoff of 0.05, and *p* value adjustment by the FDR method. *Y-axis*: Relative abundance expressed as log-transformed counts

**Fig. 6:**
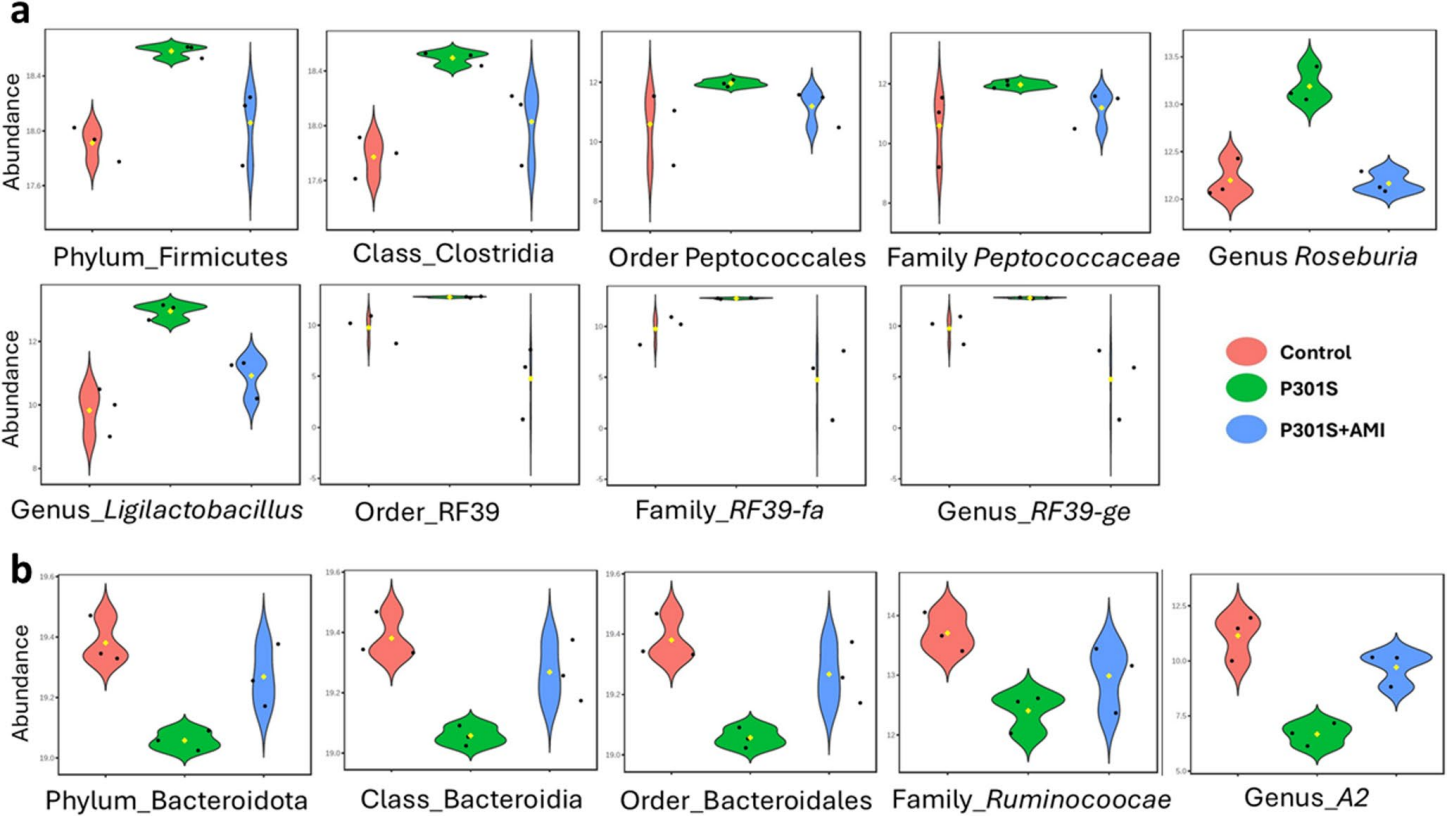
Taxa with significant differences in relative abundance among the three tested groups. Violin plots describe differential relative abundance of thirty-five significant taxa in different samples; (**a**) abundant taxa in tauopathy P301S mice, (**b**) scarce taxa in tauopathy P301S mice. The significance was identified by LEfSe analysis, with *p* value cutoff of 0.05, and *p* value adjustment by the FDR method. *Y-axis*: Relative abundance expressed as log-transformed counts

**Fig. 7:**
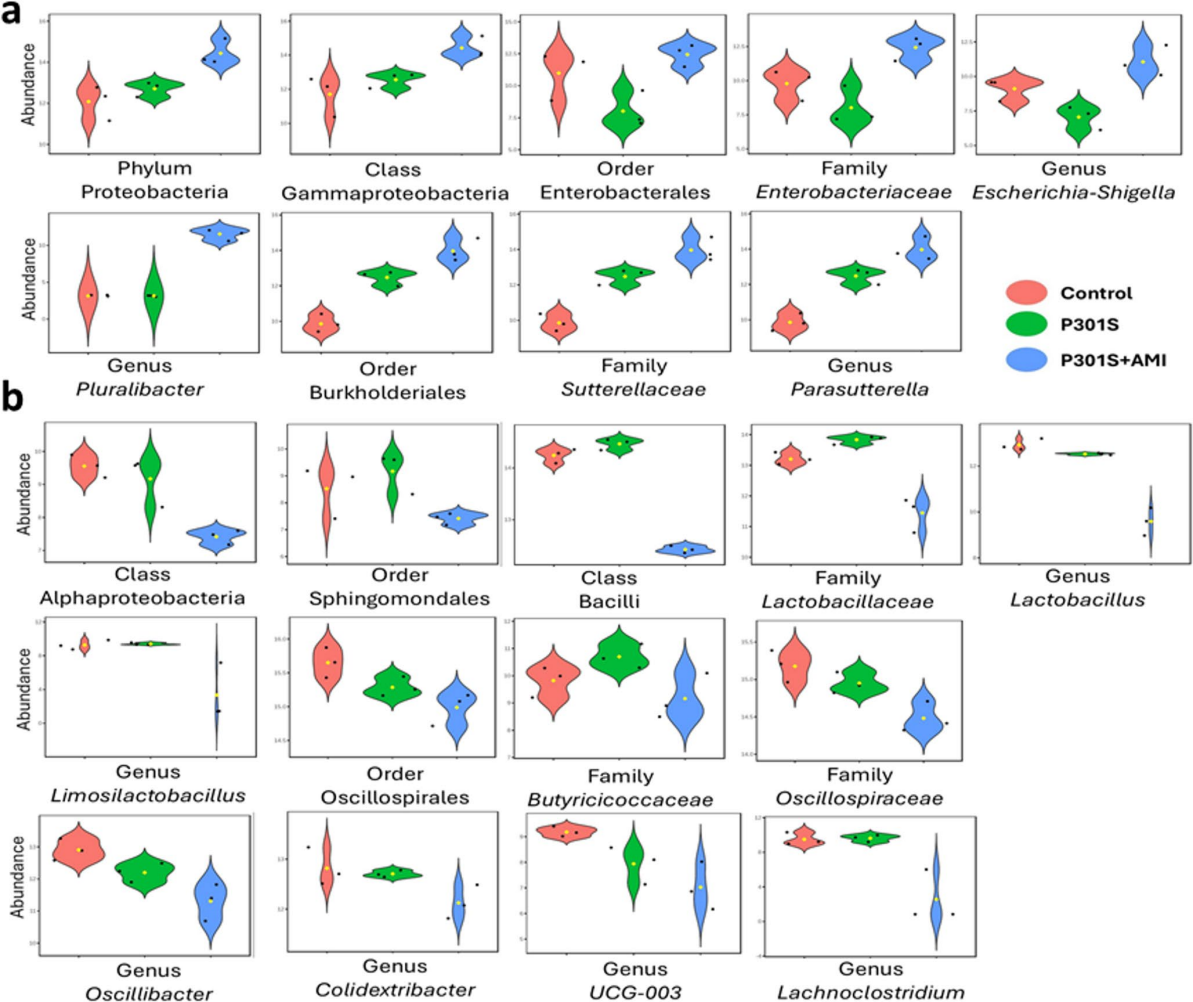
Taxa with significant differences in relative abundance among the three tested groups. Violin plots describe differential relative abundance of thirty-five significant taxa in different samples; (**a**) abundant taxa in AMI treated P301S mice, (**b**) scarce taxa in AMI treated P301S mice. The significance was identified by LEfSe analysis, with p value cutoff of 0.05, and p value adjustment by the FDR method. Y-axis: Relative abundance expressed as log-transformed counts

The analysis of microbiome profiles across the three experimental groups, using LEfSe method, revealed statistically significant differences in 35 taxa across different taxonomic levels, as illustrated in (Figs. 5, 6 and 7). Among these significantly different abundant taxa, phylum Deferribacterota (including class Deferribacteres, order Deferribacterales, family *Deferribacteraceae*, genus *Mucispirillum*), family *Rikenellaceae* (including genera *Alistipes* and *Rikenella*), family *Prevotellaceae* (genus *Alloprevotella*), phylum Campylobacterota (class Campylobacteria, order Campylobacterales, family *Helicobacteraceae*, genus *Helicobacter*), class Spirochaetia (order Spirochaetales, family *Spirochaetaceae*, genus *Treponema*), and genus *Streptococcus* were more abundant in the control group (Fig. 5a). Phylum Desulfobacterota (class Desulfovibrionia, order Desulfovibrionales), phylum Actinobacteriota (class Actinobacteria, order Bifidobacteriales, and its genus *Bifidobacterium*, and class Coriobacteriia, family *Eggerthellaceae*, genus *DNF00809*), order Lachnospirales (family *Lachnospiraceae*, genus *Lachnospiraceae_NK4A136_group*), and genus *Faecalibaculum* were less abundant also in control group (Fig. 5b).

Phylum Firmicutes (class Clostridia, order Peptococcales, family *Peptococcaceae*, and its genera *Roseburia* and *Ligilactobacillus*, and order RF39, family *RF39_fa* and genus *RF39_ge*) were more abundant in the P301S tauopathy model (Fig. 6a). Phylum Bacteroidota (including class Bacteroidia, order Bacteroidales, and family *Ruminococcaceae*, genus *A2*) were less abundant in the P301S tauopathy model (Fig. 6b).

Order Enterobacterales (Family *Enterobacteriaceae*, which include genera *Escherichia-Shigella* and *Pluralibacter*), and order Burkholderiales (which includes Family *Sutterellaceae* and genus *Parasutterella*)- both belong to Class Gammaproteobacteria- were more abundant in the AMI-treated P301S mice group (Fig. 7a). Class Alphaproteobacteria (order Sphingomondales), class Bacilli (which include Family *Lactobacillaceae* and genera *Lactobacillus* and *Limosilactobacillus*), order Oscillospirales (which include family *Butyricicoccaceae*, and genera *Oscillibacter*, *Colidextribacter*, and *UCG-003*- all belonging to family *Oscillospiraceae*), and genus *Lachnoclostridium* were less abundant in the AMI-treated P301S mice group (Fig. 7b).

#### Comparative Analysis of the Fecal Microbiome Diversity and Microbiome Profiles of P301S and AMI-Treated P301S Experimental Groups

We directly compared the untreated tauopathy model group- which depicts the pathological baseline condition without any intervention- to the AMI-treated P301S mice group- which incorporates the same pathological condition with the intervention of AMI- to assess the potential microbiome-modulating effect of the used therapeutic agent (AMI). By comparing these two groups, it is possible to assess the extent to which the treatment ameliorates or reverses the tauopathy-driven dysbiosis, providing insights into the AMI potential efficacy.

**Fig. 8:**
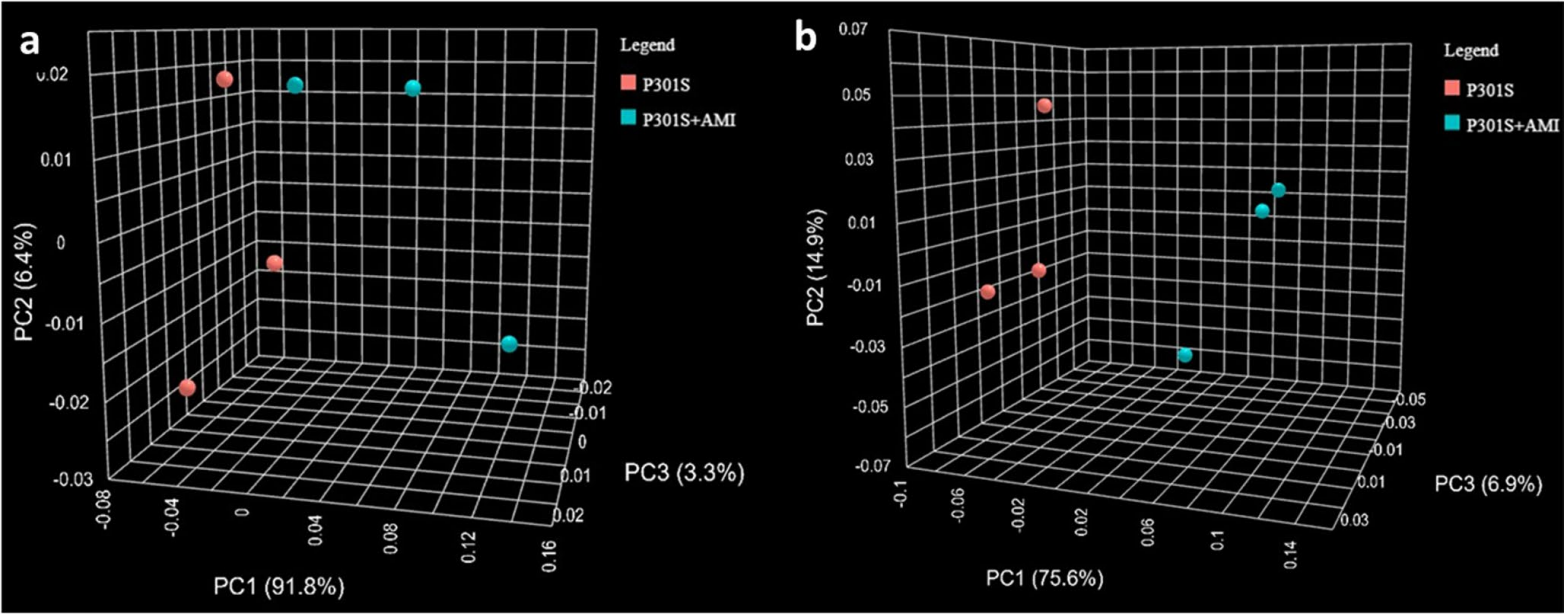
Beta-diversity, visualized by PCA of Bray–Curtis distances between different samples. (**a**) Comparison between P301S mice and AMI treated P301S mice groups at the phylum level: samples are separated into two partially-distinct clusters—PERMANOVA F-value = 10.683, R-squared = 0.72758, *p* value = 0.01, (**b**) Comparison between P301S mice and AMI treated P301S mice groups at the genus level: the samples are separated into two distinct clusters—PERMANOVA F-value = 8.0572, R -squared = 0.66825, p value = 0.01

The beta diversity, assessed through PCA of Bray–Curtis distances of all sequence variants, indicated a partial segregation between the tauopathy model group and AMI-treated P301S mice group at the phylum level, and a clear segregation into two distinct clusters at the genus level (Fig. 8), confirming the previously-detected segregation between the three different experimental groups (Fig. 4).

**Fig. 9:**
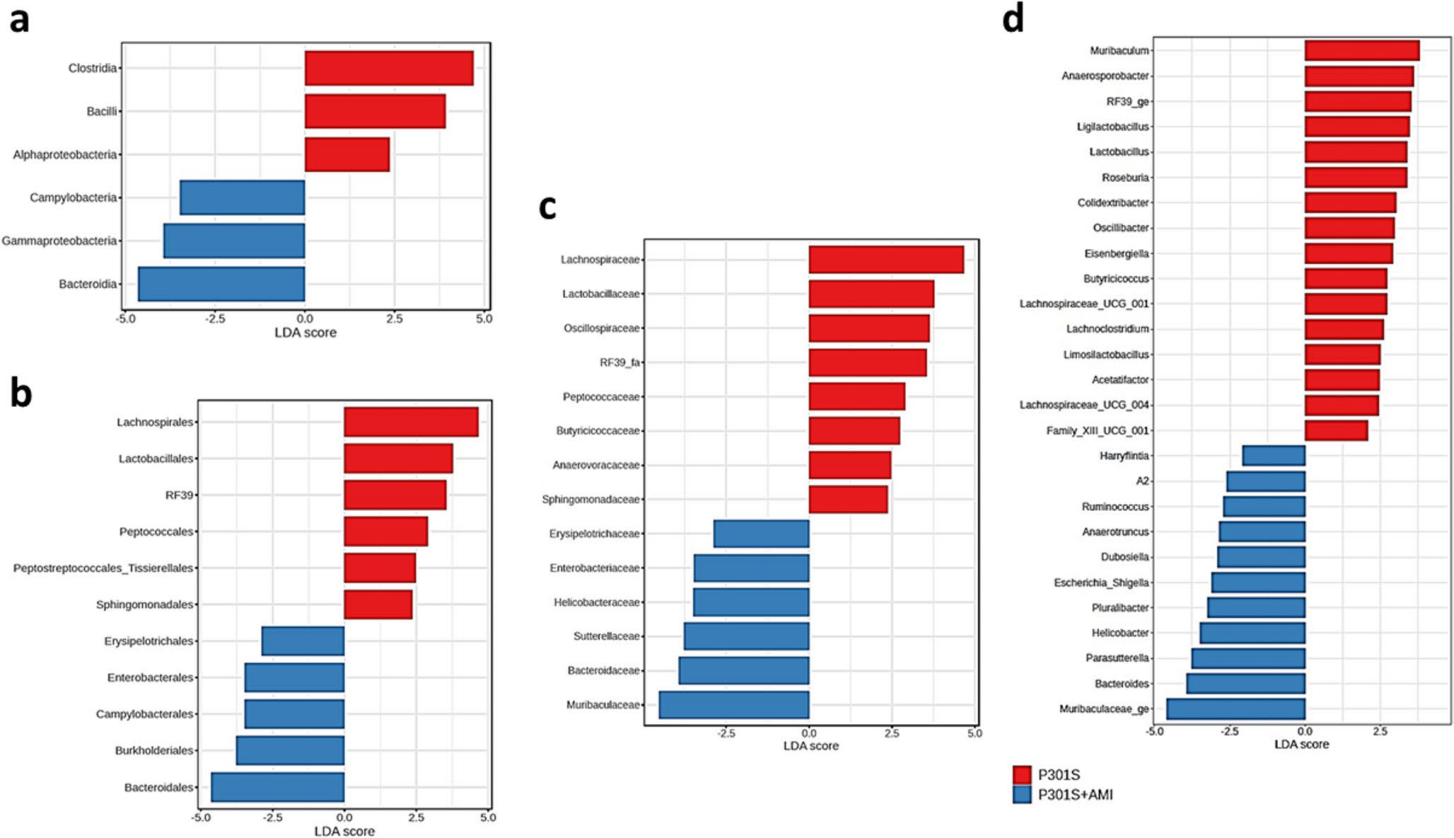
Taxa with significant differences in relative abundance between P301S mice and AMI treated P301S mice groups, the length of the bars represents the logarithm of linear discriminant analysis (LDA). Red bars represent enriched taxa in P301S mice group; blue bars represent enriched taxa in the AMI treated P301S mice group; (**a**) enriched taxa belong to the class level, (**b**) the order level, (**c**) the family level and (**d**) the genus level

We performed LEfSe analysis to identify the differences in the relative abundance in microbial taxa between the tauopathy model group and AMI-treated P301S mice group and the comparative analysis revealed statistically significant differences in several taxa across different taxonomic levels, from class to genus levels, summarized in (Fig. 9). Among the significantly different abundant taxa, class Bacteroidia (which includes order Bacteroidales, genera *Bacteroides* and *Muribaculaceae_ge*), order Erysipelotrichales (and its genus *Dubosiella*), class Gammaproteobacteria (including order Burkholderiales and its genus *Parasutterella*, and order Enterobacterales and its genera *Escherichia-Shigella* and *Pluralibacter*), and genera *Harryflintia*, *A2*, and *Anaerotruncus*-all belonging to order Oscillospirales- were significantly more abundant in the AM- treated P301S mice group (Fig. supplementary 4a).

On the other hand, class Alphaproteobacteria (including order Sphingomonadales), class bacilli (including order Lactobacillales and its genera *Lactobacillus*, *Ligilactobacillus*, and *Limosilactobacillus* and order RF39 and its genus *RF39-ge*), class Clostridia (including order Peptococcales, order Peptostreptococcales-Tissierellales and its genus Family_XIII_UCG-001, and order Lachnospirales and its genera; *Acetatifactor*,* Anaerosporobacter*, *Eisenbergiella*, *Lachnoclostridium*, *Lachnospiraceae_UCG-004*, and *Roseburia*), and genera *UCG-002*, *Colidextribacter*, *Butyricicoccus*, and *Oscillibacter*-all belonging to order Oscillospirales-were significantly less abundant in the AMI-treated P301S mice group (Fig. supplementary 4b).

### Effects of AMI Treatment on Neurodegeneration and Histopathological Changes in Brain and Distal Colon of P301S Mice

**Fig. 10:**
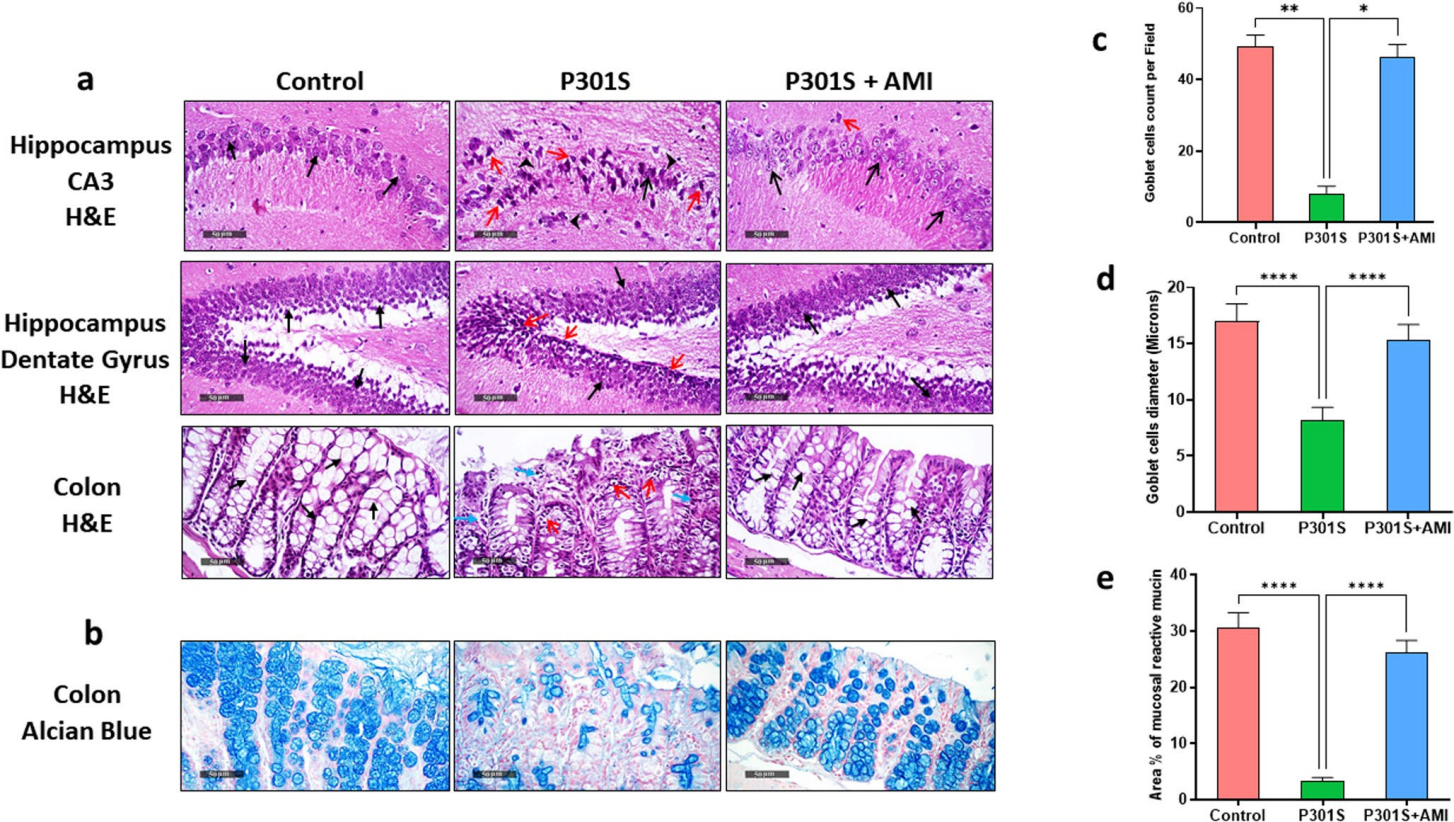
Effect of AMI treatment on tauopathy induced histopathological changes represented by (**a**) photomicrographs of H&E stained two distinct regions of hippocampal sections (hippocampus CA3 region and hippocampus Dentate Gyrus region) and H&E-stained distal colon sections in three experimental groups. (400X scale bar = 50 μm). (**b**) Photomicrographs of Alcian blue stained distal colon sections. (400X scale bar = 50 μm). (**c**) Goblet cells count in distal colon. (**d**) goblet cells diameter in distal colon. (**e**) Percentage of mucin area in distal colon. All data are presented as a mean ± SD. (*n* = 6). One-way analysis of variance (ANOVA) was used to determine statistical significance, followed by Tukey’s multiple comparison analysis except for the goblet cells count in distal colon which was tested using Kruskal–Wallis test to determine statistical significance, followed by Dunn’s multiple comparison test. A p-value below 0.05 was considered significant where *p ≤ 0.05, **p ≤ 0.005, ***p ≤ 0.001, ****p ≤ 0.001

The effects of AMI treatment on the histopathological alterations associated with tauopathy in brain was explored by investigation of H&E-stained sections of hippocampus; Cornu Ammonis 3 (CA3) and Dentate gyrus regions. As shown in (Fig. 10a), Histopathological examination of P301S mice showed pronounced disorganization of CA3 pyramidal neurons with significant loss, extensive number of degenerated and shrunken neurons which had ill-defined subcellular details (red arrow) associated with moderately vacuolized intercellular brain matrix, perineuronal edema, minimal numbers of apparent intact neurons (black arrow) besides extensive reactive microglial cells infiltration (arrowhead). In contrast, normal histological features of CA3 region including widespread number of apparent intact pyramidal neurons with clearly distinct subcellular and nuclear details (black arrow) along with minimally degenerative alterations with intact intercellular matrix were detected in control group. Further, hippocampal CA3 region sections from P301S group received AMI showed evidently neuroprotective effects with few sporadic numbers of neuronal degenerative modifications (red arrow) and higher prevalence and number of apparent intact pyramidal neurons (black arrow) without abnormal cellular infiltrates. Regarding the Dentate gyrus region, H&E-stained sections from P301S mice showed mild to moderate records of pyknotic inner granule cells (red arrow) that alternated with apparent intact cells (black arrow) along with minimal abnormal infiltration of glial cells. However, control group and AMI-treated P301S mice showed normally organized histological structures of hippocampal layers involving layer of granule cells at the apexes and blades of the hippocampus with distinct subcellular details (arrow) and a hilar region with few glial cells’ infiltration and occasional figures of degenerated cells. Based on the regulation of the bidirectional gut-brain axis, the impact of AMI treatment on histopathological alterations in the distal colon correlated with tauopathy was also investigated. As shown in (Fig. 10a), H&E-stained distal colon sections from the control group showed a normally organized structure of distal colon wall that shows abundant goblet cells (black arrow) and covering epithelium with intact colonic crypts and normal submucosa and outer muscular coat. On the other hand, sections from P301S mice demonstrated marked mucosal disorganization and loss of morphological features of colonic wall including focal superficial erosions, minimal glandular goblet cells and vacuolar degenerative changes with nuclear pyknosis of enterocytes (red arrow) accompanied with mild to moderate mucosal inflammatory cells infiltrates (blue arrow). Remarkably, sections from AMI-treated P301S mice showed obvious improvement of colonic wall morphologies with abundant figures of apparent intact epithelium lining as well as the glandular elements of mucosa with a notable rise in number of mature goblet cells (black arrow) and minimal inflammatory cells of mucosa/submucosa were observed in the majority of examined sections.

Furthermore, Alcian blue staining was performed to assess the goblet cells count and diameter in addition to percentage of mucin area in distal colon. As shown in (Fig. 10b-e**)**, P301S mice showed significant reduction in goblet cells count, goblet cells diameter and percentage of mucin area by 84% (*P* = 0.0017), 52% (*P* < 0.0001, F _(2,15)_ = 70.38) and 89% (*P* < 0.0001, F _(2,15)_ = 307.9) respectively comparing to control group. However, AMI treatment in P301S mice remarkably increased the count of goblet cells, diameter of goblet cells and percentage of mucin area by 6.4 folds (*P* = 0.0474), 1.9 folds (*P* < 0.0001, F _(2,15)_ = 70.38) and 8.4 folds (*P* < 0.0001, F _(2,15)_ = 307.9) respectively comparing to tauopathy model. These changes in the intestinal barrier structure suggest that AMI improves intestinal homeostasis in tauopathy.

### Effects of AMI Treatment in P301S Mice on Barrier Integrity and LPS Levels in Serum

**Fig. 11:**
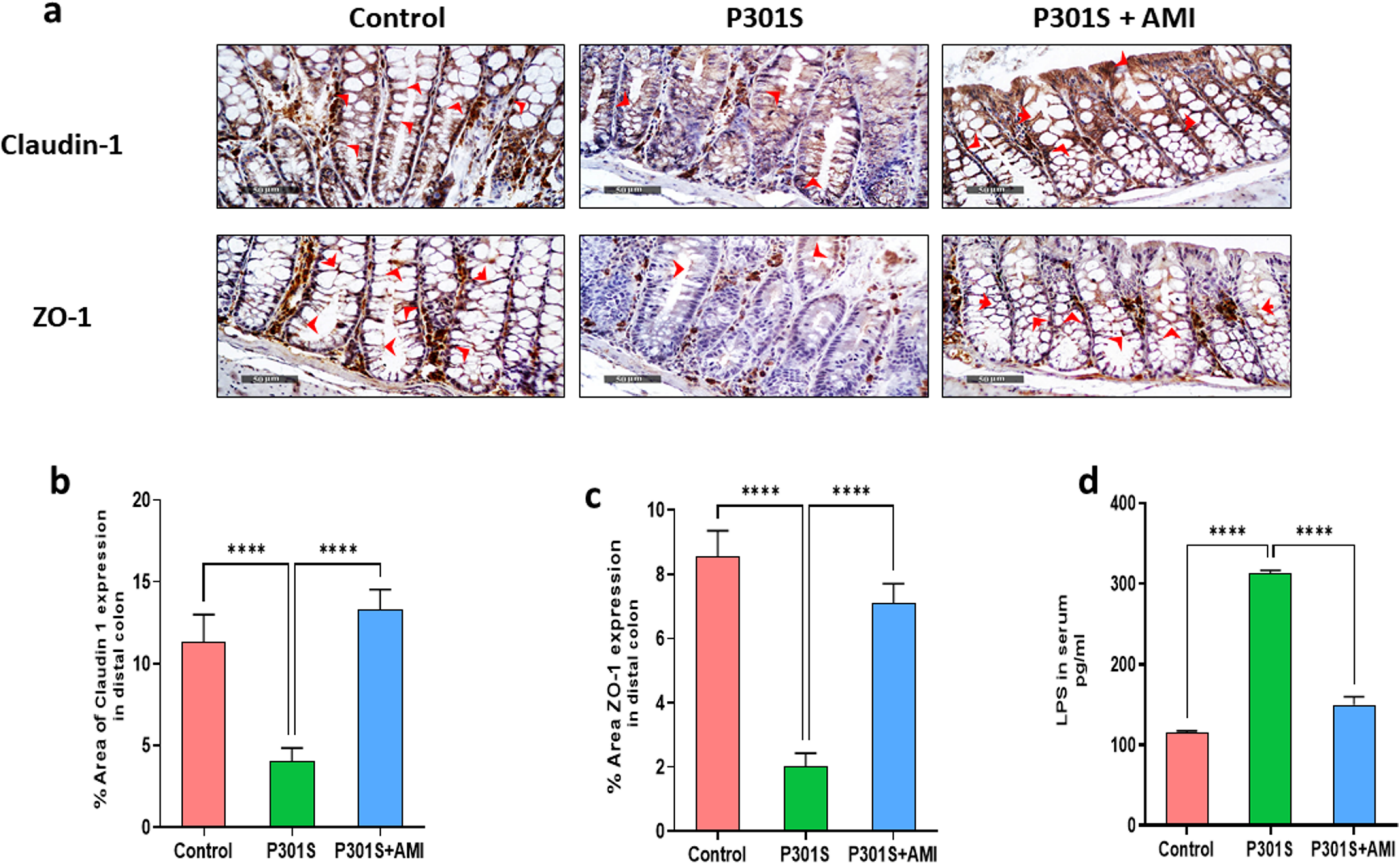
Effect of AMI treatment on intestinal barrier disruption and subsequent serum LPS levels associated with tauopathy in P301S mice. (**a**) Photomicrographs showing the expression of claudin-1 and ZO-1 tight junction proteins in distal colon in the three experimental groups (400X scale bar = 50 μm). (**b**) A graph showing the percentage area of immunostaining for claudin-1 tight junction protein in distal colon in the three experimental groups. (**c**) A graph showing the percentage area of immunostaining for ZO-1 tight junction protein in distal colon in the three experimental groups. (**d**) serum LPS levels in the three experimental groups All data are presented as a mean ± SD. (*n* = 6). One-way analysis of variance (ANOVA) was used to determine statistical significance, followed by Tukey’s multiple comparison analysis. A p-value below 0.05 was considered significant where *p ≤ 0.05, **p ≤ 0.005, ***p ≤ 0.001, ****p ≤ 0.001

Tauopathies are associated with changes in the gut barrier, that may let the bacteria and bacterial products to cross the mucosal barrier of intestine causing bowel motor dysfunctions, then reach bloodstream and trigger central and systemic immune and inflammatory responses. (Pellegrini et al. 2023) Here, the impact of AMI on maintaining the integrity along with intestinal barrier organization was evaluated. Firstly, we investigated the expression levels of the membrane adaptor protein ZO-1and tight junction protein claudin-1 to assess the intestinal membrane integrity. As shown in (Fig. 11a-c), P301S mice showed significant downregulation of expression level of claudin-1 and ZO-1 by 63% (*P* < 0.0001, F _(2,15)_ = 85.7) and 76% (*P* < 0.0001, F _(2,15)_ = 185.9) respectively comparing to control group which significantly upregulated by AMI treatment by 3.4 folds (*P* < 0.0001, F _(2,15)_ = 85.7) and 3.7 folds (*P* < 0.0001, F _(2,15)_ = 185.9) respectively when compared to P301S mice. Further, we evaluated the circulating levels of LPS in serum, which is negatively correlated with intestinal barrier integrity (Buford 2017). In P301S mice, LPS serum levels were significantly enhanced by 2.7 folds (*P* < 0.0001, F _(2,15)_ = 1679) when compared with control group, suggesting an impairment of intestinal epithelial barrier permeability associated with tauopathy. However, treatment with AMI in P301S mice was able to restore LPS levels to reach levels approximately like control group by significant reduction by 52% (*P* < 0.0001, F _(2,15)_ = 1679), as compared with P301S mice (Fig. 11d).

### Effects of AMI Treatment on P-tau/PP2A/BDNF in the Brain of P301S Mice

**Fig. 12:**
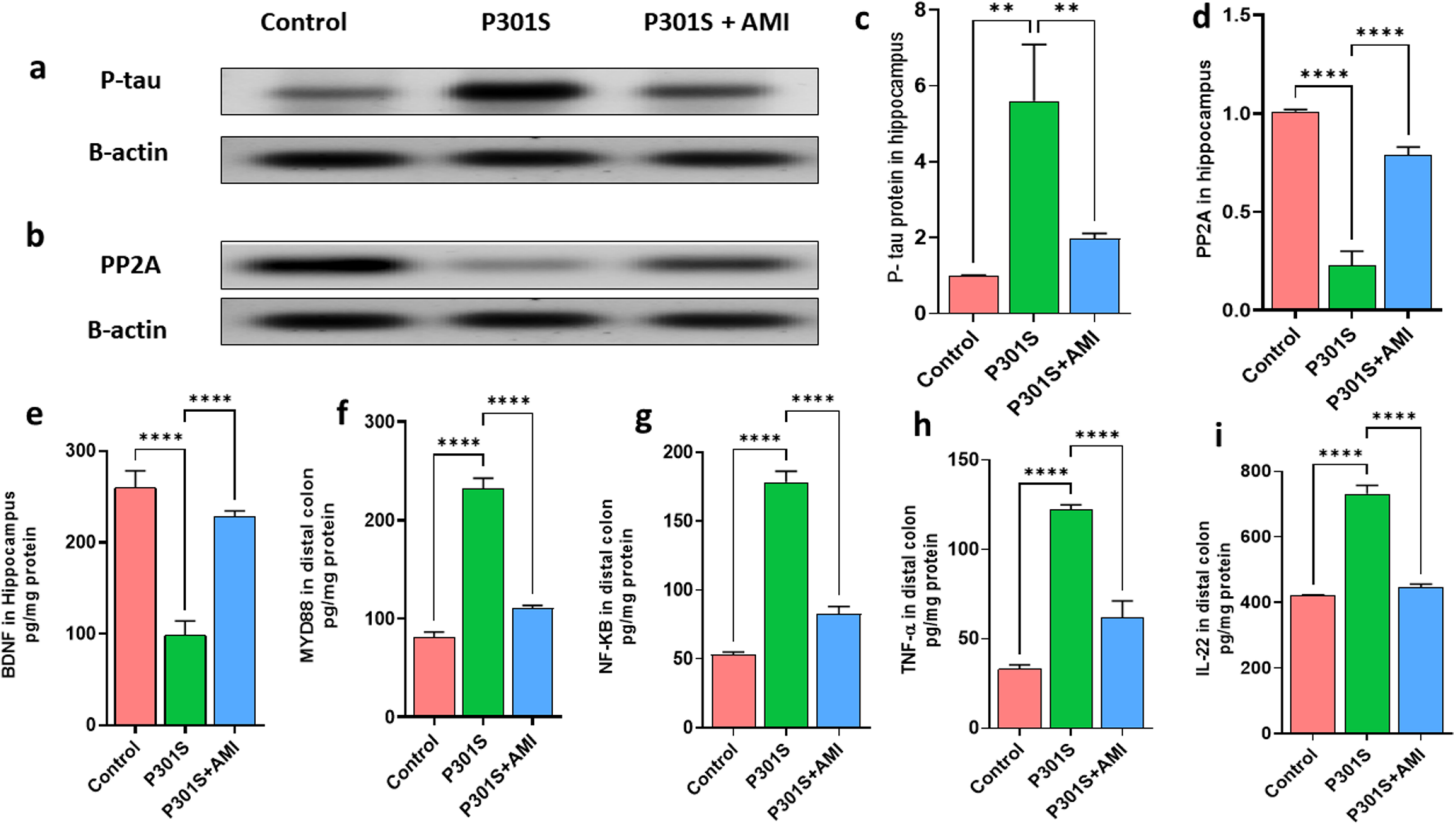
Effect of AMI treatment on tauopathy related markers in hippocampus and inflammatory markers in distal colon of P301S mice. Panels (**a**&**c**) represent western blot of phosphorylated tau in hippocampus and its quantitation (*n* = 3). Panel (**b**&**d**) Represents western blot of PP2A in hippocampus and its quantitation(*n* = 3). (**e**) Hippocampal content of BDNF(*n* = 6). (**f**) Distal colon content of MYD88 (*n* = 6). (**g**) Distal colon content of NF-KB (*n* = 6). (**h**) Distal colon content of TNF-α (*n* = 6). (**i**) Distal colon content of IL-22 (*n* = 6). All data are presented as a mean ± SD. One-way analysis of variance (ANOVA) was used to determine statistical significance, followed by Tukey’s multiple comparison analysis. A p-value below 0.05 was considered significant *p ≤ 0.05, **p ≤ 0.005, ***p ≤ 0.001, ****p ≤ 0.001

Levels of P-tau were investigated in the hippocampus using Western blotting to explore how AMI affects P-tau deposition and pathology in P301S mice besides modulation of gut dysbiosis, intestinal membrane integrity and SPL metabolism. Tau hyperphosphorylation in p301s mice is a characteristic feature (Allen et al. 2002) as shown in tauopathy model with significant increase in levels of P-tau protein by 5.6 folds (*P* = 0.0015, F _(2,6)_ = 23.46) comparing with control group. However, AMI treated P301S mice showed significant decrease in P-tau protein level when compared to tauopathy model by 62% (*P* = 0.0052, F _(2,6)_ = 23.46) (Fig. 12a and c). PP2A is a major phosphatase enzyme involved in tau dephosphorylation (Martin et al. 2013; Wei et al. 2020). To investigate the effect of AMI treatment in P301S mice on PP2A levels and consequent reduction in P-tau levels, PP2A protein levels were assessed using western blotting which showed significant decreased levels of PP2A in P301S mice by 78% (*P* < 0.0001, F _(2,6)_ = 220.5) comparing to control group. However, AMI treated P301S mice showed significant upregulation in PP2A levels by 3.7 folds (*P* < 0.0001, F _(2,6)_ = 220.5) when compared with tauopathy model (Fig. 12b and d). Also, BDNF which can regulate activity-dependent synaptic plasticity and psychiatric disorders (Leal et al. 2017) was assessed. P301S mice showed significant decrease in levels of BDNF by 61% (*P* < 0.0001, F _(2,15)_ = 215.2) comparing with control group which increased significantly with AMI treatment by 2.4 folds (*P* < 0.0001, F _(2,15)_ = 215.2) comparing with P301S mice (Fig. 12e).

### Effects of AMI Treatment in P301S Mice on Distal Colon Inflammation

Considering the strong correlation between ASM and inflammation (Maceyka and Spiegel 2014), we want to investigate the effect of AMI treatment on tauopathy mediated gut inflammation. So, MYD88, NF-KB, TNF-α and IL-22 levels were measured. Notably, LPS-induced TLR4 activation can trigger the downstream proinflammatory pathway of the MyD88/NF-κB pathway and thereafter release a lot of inflammatory mediators as TNF-α and IL-22 resulting in intestinal injury (Cong et al. 2018). P301S mice showed elevated level of MYD88, NF-KB, TNF-α and IL-22 by 2.9 folds (*P* < 0.0001, F _(2,15)_ = 770.9), 3.5 (*P* < 0.0001, F _(2,15)_ = 794.5), 3.7 (*P* < 0.0001, F _(2,15)_ = 385.6) and 1.7 (*P* < 0.0001, F _(2,15)_ = 668.8) respectively comparing to the control group. While AMI treated P301S mice demonstrated significant decrease in the levels of MYD88, NF-KB, TNF-α and IL-22 by 52% (*P* < 0.0001, F _(2,15)_ = 770.9), 54% (*P* < 0.0001, F _(2,15)_ = 794.5), 49% (*P* < 0.0001, F _(2,15)_ = 385.6) and 39% (*P* < 0.0001, F _(2,15)_ = 668.8) respectively when compared to tauopathy model (Fig. 12f-i).

## Discussion

The gut-brain axis provides systemic physiological coordination by acting as a bidirectional communication channel between the regulatory centers of the structural brain and the peripheral activity of the gut. (Martin et al. 2018; Cryan et al. 2019). Recent studies have emphasized the GM as a dynamic ecosystem of trillions of microorganisms, significantly impacting and regulating human health throughout life (Yatsunenko et al. 2012; Merlo et al. 2024). The GM composition, modulated by a range of factors including diet, age, metabolic state, genetics, and stress (Bates 2017), has a significant influence on systemic metabolism (Visconti et al. 2019), immunity (Belkaid and Hand 2014), and the central nervous system (Ma et al. 2019). Central to this interplay is intestinal homeostasis, a dynamic equilibrium maintained through the activity of microbial communities and their metabolites, maintenance of mucosal integrity, and immune regulation. Alterations in this equilibrium have been correlated with the development and progression of AD (Chen et al. 2022; Borsom Emily et al. 2023). Additionally, intestinal dysbiosis impairs the integrity of the intestinal barrier, resulting in increased permeability and intestinal leakage, which permits the excretion of neurotransmitters, cytokines, chemokines, and gut-derived metabolites into the blood (Liang et al. 2024), further aggravating BBB dysfunction, which modulates the development and progress of AD (Uchida et al. 2023; Aburto and Cryan 2024). Notably, both the function and the signaling pathways of the vital proteins are influenced by the lipid-rich microenvironment of cell membranes (Rohr et al. 2020), and this emphasize the crucial role of the GM in transforming dietary lipids into bioactive metabolites that modulate systemic immunity and host metabolism (Chadaideh and Carmody 2021).

Ceramide and ASM have been shown to have a significant role in receptor signaling in previous studies. As a lysosomal hydrolase, the glycoprotein ASM acts accelerates the process of lysosomes’ breakdown of sphingomyelin into ceramide and phosphorylcholine (Carpinteiro et al. 2020). Ceramide is produced in the outer leaflet of the cell as a result of the activity of ASM (Gulbins and Kolesnick 2003; Carpinteiro et al. 2020). Many antidepressants, including amitriptyline, functionally inhibit ASM (FIASMA) action through displacing ASM from lysosomal membranes, releasing it into the lysosomal lumen, and partially degrading it (Gulbins et al. 2013; Gulbins et al. 2018; Carpinteiro et al. 2020). Several investigations have found elevated amounts of ceramide and activity of various SPL-metabolizing enzymes in the brain tissue of patients with AD (Crivelli et al. 2024) (Lee et al. 2014) leading to a variety of pathological features such as cell apoptosis (Smith and Schuchman 2008), neurogenesis loss (Gulbins et al. 2013), BBB leakage**(**Park et al. 2018), and inflammation (Chung et al. 2016; Bai and Guo 2017), indicating that ASM inhibition could be an important therapeutic target for addressing AD’s neuropathological features (Park et al. 2020; Park et al. 2022). But very little data is known about the role of intestinal ASM in tauopathies. Considering the importance of a balanced GM homeostasis for intestinal barrier function and its correlation with SPL metabolism. Therefore, the purpose of this study was to investigate how AMI, as one of the FIASMA drugs affects gut dysbiosis, levels of ceramides in the colon, associated colonic inflammation, intestinal barrier disruption and the subsequent effects on tauopathy related markers in the brain through the bidirectional gut-brain axis.

The symbiotic relationship between the GM and its host profoundly influences host physiology, including brain function under both normal and pathological conditions (Chu et al. 2019; Zhu et al. 2024) GM dysbiosis in AD has been linked to altered behavior, neuronal ultrastructure, and BBB integrity (Hoyles et al. 2021; van Olst et al. 2021). In this study, P301S tauopathy model mice exhibited marked dysbiosis, characterized by reduced microbial diversity -demonstrated by genus-level analysis using Shannon, Simpson, and Fisher indices-, and microbiome compositional shifts compared to the control group—a pattern mirroring previous findings that linked gut dysbiosis to neurodegenerative diseases either in mice (Borsom Emily et al. 2023), or in humans (patients with AD) (Vogt et al. 2017; Liu et al. 2019; Hung et al. 2022). Remarkably, AMI treatment restored GM diversity, aligning with earlier studies showing similar effects in chronic stress models (Zhang et al. 2021).

In-depth taxonomic analysis, using LEfSe, revealed significant changes in 35 taxa across various levels. P301S tauopathy mice exhibited a phylum-level higher abundance of Firmicutes, alongside low levels of Bacteroidota, which have been demonstrated as intestinal barrier-protective (Hsiao et al., 2013); Cattaneo et al. 2017; Zhuang et al. 2018; Zhan et al. 2023). This relatively higher Firmicutes-to-Bacteroidota ratio in P301S mice mirrors microbiota alterations observed in other neurodegenerative conditions such as AD **(**Vogt et al. 2017; Zhan et al. 2023), and might reflect pro-inflammatory microbial dynamics, aggravating tauopathy progression. Class-level lower relative abundance of Bacteroidia and a higher relative abundance of Clostridia (which has been positively correlated with cerebral P-tau protein (Sun et al. 2019) were observed, alongside family-level enrichment of *Peptococcaceae* and relative depletion of *Ruminococcaceae* (butyrate-producer (Fu et al. 2019)—a putative biomarker of AD progression and memory impairment (Shen et al. 2017; Vogt et al. 2017; Zhuang et al. 2018; Yang et al. 2023). At genus level, there was a notable enriched abundance of *Roseburia*, and *Ligilactobacillus* genera and a relative depletion in the abundance of *Lachnospiraceae bacterium A2*, which is associated with intestinal anti-inflammatory T-regulatory cells and negatively correlated with repetitive behaviour in autism (van de Wouw et al. 2021). Of note, *Peptococcaceae* and *Roseburia*, enriched in P301S mice, were identified as mucin degraders, potentially contributing to barrier dysfunction (Amaretti et al. 2019; Glover et al. 2022). Also, Lactobacillales order (including *Ligilactobacillus*, and *Limosilactobacillus*, enriched in P301S mice), was found to be among the abundant orders in patients with AD (Zhan et al. 2023). Conversely, control mice exhibited higher abundances of taxa such as phylum Deferribacterota (including genus *Mucispirillum*), *Rikenilla*, *Alloprevotella* and *Altistipes* which are negatively correlated with AD and tau protein in brain (Shen et al. 2017; Hung et al. 2022; Troci et al. 2023). Also, control group had a relatively lower abundance of Desulfobacterota which is known to be butyrate metabolizing (Suzuki et al. 2010), and *Bifidobacterium* which positively correlate with AD and tau protein in the brain (Shen et al. 2017; Saji et al. 2019; Hung et al. 2022; Sheng et al. 2022; Ferreiro et al. 2023). Moreover, *Bifdobacterium* genus is capable of converting nitrate and nitrites into nitric oxide leading to exacerbation of AD disease (Das and Ganesh 2023), and is positively correlated with elevated gut ceramides in depression (Wang et al. 2024a). Remarkably, *Bifidobacterium* strains lacking the SPL synthesis enzyme are able to utilize and import SPL to produce dihydroceramide (Lee et al. 2021), highlighting GM-SPL crosstalk.

AMI administration in the P301S tauopathy model resulted in a significant shift in the gut microbial communities, as indicated by beta diversity analysis. PCA plots, based on Bray–Curtis metrics, demonstrated a clear visual separation among experimental groups, with a distinct segregation at both phylum and genus levels. Notably, the distinct clustering of P301S and AMI-treated P301S groups, especially at the genus level (Fig. 8), highlights the microbiome-modulating potential impact of amitriptyline, which partially reversed the tauopathy-driven dysbiosis observed in P301S group. This finding aligns with the growing evidence of the gut microbiota-modulating effects of the targeted interventions —whether pharmacological or dietary—, and thereby their profound impact on the host health and the progression of many diseases (Attia et al. 2023). A notable enrichment in *Dubosiella* was found in AMI-treated P301S mice than in the untreated group, an observation that aligns with prior findings that associate *Dubosiella* enrichment with the attenuation of AD progression through the anti-inflammatory palmitoleic acid biosynthesis, offering a mechanistic link between microbial modulation and neuroprotection (Chen et al. 2024b). AMI-treated exhibited a lower relative abundance-compared to the untreated P301S group- of taxa previously known to be positively correlated with neurodegenerative diseases like AD (Saji et al. 2019; Das and Ganesh 2023; Ferreiro et al. 2023); Specifically *Lactobacillus*, *Lachnoclostridium* -which are also linked to Crohn’s diseases **(**Alsulaiman et al. 2023), and p-tau protein aggregation in the brain **(**Borrego-Ruiz and Borrego 2024)- as well as *Oscillibacter*, *Oscillospiracea UCG-003*, *Colidextribacter* genera, and mucin degrader taxa that contribute to mucosal barrier dysfunction such as Peptococcales, Peptostreptococcales-Tissierellales orders, *Oscillospiracea* family, and *Roseburia* genus (Amaretti et al. 2019; Glover et al. 2022). AMI-treated P301S group had relatively higher abundance of *Harryflintia*, a beneficial butyrate-producing genus of family *Ruminococcaceae* (Fu et al. 2019), and *Dubosiella*. AMI-treated P301S group also demonstrated an enrichment of *Parasutterella*, a genus which was enriched in the healthy control group, and a notable low abundance of *Butyricicoccus*, a genus associated with abstraction impairment in patients with AD in a recent study (Chen et al. 2024a). Similarly, Alphaproteobacteria class, particularly Sphingomonadales order, which are associated with autoimmunity (Mohammed and Mattner 2009), were relatively depleted in AMI-treated P301S. Notably, the cell wall of Sphingomonadales (belonging to Alphaproteobacteria class) contains glycosphingolipids (GSLs)- a kind of SPL consisting of a ceramide backbone connected to a carbohydrate group- instead of the usual LPS (Kawahara et al. 1999; Kawahara et al. 2000). GSLs of several bacterial strains belonging to the order Sphingomonadales can be detected by natural killer T cells (Mattner et al. 2008; Kitahata et al. 2021). GSLs are a kind of SPL consisting of a ceramide backbone connected to a carbohydrate group. ASM converts sphingomyelin to ceramide, which can then be transformed into GSLs such as gangliosides or cerebrosides in the presence of certain enzymes. GSLs breakdown in the lysosome needs hydrolyzing enzymes such as β-galactocerebrosidase (GALC) and lipid-binding saposin protein. The saposin protein is necessary for GALC to digest lipids, and deficiencies in either of these proteins cause serious neurological disorders. Remarkably, the catalytic domains in ASM and GALC are located similarly to the saposin protein (Hill et al. 2018), indicating that FIASMA can affect GSLs and the subsequent abundance of Alphaproteobacteria class.

The GM has significant effects on the metabolism of the host, playing a pivotal role in modulating lipid metabolism and maintaining the integrity of the intestinal mucosal barrier, thereby serving as an important regulator of the gut-brain axis (Lynch and Pedersen 2016; Buford 2017; Long-Smith et al. 2020; Fan and Pedersen 2021). Bacteroidota of the mammalian GM are exceptional in their ability to produce SPL (Brown et al. 2019; Johnson et al. 2020). In mice, a lack of *Bacteroides*-derived SPL induced intestinal inflammation and increased host ceramide levels (Brown et al. 2019). In our study, P301S mice had a significantly lower abundance of Bacteroidota and a significant increase in levels of eight types of ceramides- potential contributors to neuronal death (Mandik and Vos 2021)- compared to the control group, in addition to a significant increase in ASM activity in the distal colon when compared to the control group. In patients diagnosed with inflammatory bowel disease (IBD), there was a notable decline in microbial SPL, while host-derived ceramides emerged as the predominant elevated metabolites (Brown et al. 2019). These observations pointed to a strong relationship between microbial and host SPL, as demonstrated by a previous study found that sphinganine produced by *Bacteroides* may enter the host epithelial cells and integrate with SPL metabolic pathways, changing the sphingolipidome of host (Johnson et al. 2020). In our study, AMI treatment significantly lowered the level of seven types of ceramides in P301S mice. This reduction was associated with a substantial decrease in ASM activity in the distal colon, alongside a markedy lower relative abundance of *Lactobacillus*, *Lachnoclostridium*, *Oscillibacter*, *Oscillospiracea UCG-003*,* Colidextribacter*, and Sphingomonadales. Collectively, our results underscored the importance of the GM –metabolites–brain axis interaction in regulating the potential neuroprotective effect of AMI.

The intestinal barrier plays an important role in intestinal homeostasis (Allaire et al. 2018). Tight junctions are an essential junctional complex composed of tight junction proteins, involving transmembrane proteins like occludin and claudin-1, as well as the adaptor protein of peripheral membrane ZO-1. These proteins are crucial for maintaining the intestinal barrier’s permeability and integrity (Odenwald and Turner 2017). Intestinal permeability increases when tight junction proteins are reduced (Allam-Ndoul et al. 2020).Interestingly, P301S mice in this study showed downregulation in ZO-1 and claudin-1expression levels which significantly restored after AMI treatment. In a previous research, intestinal ZO-1 and claudin-1 concentrations were markedly reduced in a mouse model of AD. (He et al. 2023). It was previously demonstrated that the ASM/ceramide system is implicated in the degradation of endothelial cell tight junction proteins by a significant reduction in ZO-1 expression, which was totally prevented by AMI pretreatment (Becker et al. 2018). In addition to serving as a protective barrier, the mucus layer covering the GI tract’s luminal surface is produced by goblet cells, which is essential for preserving the integrity of the enteric barrier (Breugelmans et al. 2022). Our findings demonstrated the decrease in goblet cell count and number in addition to mucin area in P301S mice which come in line with findings of a previous study on mouse model of AD (Wang et al. 2024b). On the contrary, AMI treatment in P301S mice restored the membrane integrity as indicated by increased count as well as size of goblet cells in addition to mucin area as previously described of protective effect of AMI on goblet cells and increasing the expression of tight junction proteins in experimental model of colitis (Zeng et al. 2024).

Inflammation-induced disruption of intestinal barrier has an important role in controlling intestinal permeability (Pellegrini et al. 2020). Inflammatory response and glial reactivity can impact the effect of GM dysbiosis on AD pathogenesis (Chen et al. 2022). Recent research suggests that ASM activity plays a crucial role in inflammatory processes (Maceyka and Spiegel 2014). In this regard, it has been discovered that ASM inhibition lowers the production of inflammatory cytokines mediated by LPS and protects mice with chemically induced colitis from disease pathology (Sakata et al. 2007; Xiong et al. 2019). TNF-α and other cytokines activate sphingomyelinase, leading to a rise in ceramide levels across tissues and cell lines (Hannun and Linardic 1993). Furthermore, certain bacterial toxins, such as LPS, raise ceramide levels via a TLR4-dependent mechanism (Fischer et al. 2007). Modulating the TLR4/MYD88/NF-κB signaling pathway can mitigate gut dysbiosis, intestinal membrane disruption, LPS-induced neuroinflammation and impaired cognition in AD mouse models (Zhou et al. 2019; Guo et al. 2020; Su et al. 2023). Resident intestinal inflammation as a result of GM dysbiosis can alter intestinal barrier integrity and promote permeability, a condition known as leaky gut (Zhou et al. 2019; Guo et al. 2020). Therefore, pro-inflammatory bacterial products such as cytokines and LPS pass the impaired barrier into the blood, resulting in systemic inflammation (Buford 2017), BBB breakdown, and neurodegeneration (Friedland 2015; Zhou et al. 2020). In this research, we noticed that AMI significantly reduced NF-κB, MYD88, TNF-α and IL-22 levels in distal colon and serum LPS of P301S mice, indicating that AMI may improve neuroinflammation by regulating the gut-brain axis.

Accordingly, amount of insoluble NFTs in the hippocampus is the main pathologic hallmark of tauopathies (Allen et al. 2002; Geschwind 2003; Delobel et al. 2008). P301S mice have an accumulation of P-tau protein (Wang and Liu 2008; Hanger et al. 2009).We wanted to explore whether AMI treatment mediated modulation of gut dysbiosis, SPL metabolism, intestinal membrane permeability and peripheral gut inflammation has an impact on P-tau regulation. Notably, blood SPL concentrations are correlated with cerebrospinal fluid p-tau levels and cognitive decline, highlighting the possibility of SPL as an early biomarker of AD (Varma et al. 2018). Interestingly, AMI significantly attenuated P-tau in P301S mice which suggests its neuroprotective effect against tauopathy development. Kinases and phosphatases work in balance to regulate tau phosphorylation and dephosphorylation. Similarly, various phosphatases, like PP2A, are crucial for the proper control of tau hyperphosphorylation along with dephosphorylation (Taleski and Sontag 2018). PP2A is considered the major phosphatase in the brain for the dephosphorylation of P-tau (Martin et al. 2011; Wang et al. 2019). A prior research confirmed the increased levels of P-tau protein and lowered activity of PP2A in P301S mice (Goedert et al. 2000). These findings were proven in the current study by significant downregulation of PP2A level in P301S. However, AMI administration in P301S mice significantly increases the levels of PP2A suggesting the neuroprotective effect of AMI against tauopathy. Moreover, the hippocampus is capable of neurogenesis, or the continuous production of new neurons (Altman and Das 1965), that plays a major role in learning and memory (Kim et al. 2008) BDNF modulates activity-dependent synaptic plasticity and psychiatric conditions**(**Björkholm and Monteggia 2016; Leal et al. 2017), Studies have revealed that the GM upregulates hippocampus BDNF (O’Leary et al. 2018). Notably, BDNF was found to be significantly downregulated in P301S mice which upturned significantly by AMI treatment as previously reported in AD mouse model (Roushdy et al. 2024). Moreover, examination of microphotograph of H&E-stained sections of hippocampus region was carried out which showed severe neuronal degeneration and higher records of reactive microglial cells infiltrate in P301S mice when compared to control group. In contrast, hippocampal sections from P301S mice received AMI showed reduction in neuronal degeneration with higher prevalence of apparent intact pyramidal neurons as previously recorded of protective effect of AMI on hippocampal structure in chronic stress model (Roushdy et al. 2024). In particular, the GM is required for the proper development of hippocampus and microglial cell morphology (Luczynski et al. 2016). Liu et al. found that GM deficit changed dendritic signaling integration in the Cornu Ammonis1 region (Liu et al. 2022). The P301S transgenic mice used in this research are distinguished by their development of tau pathology and NTFs at 3 months of age. (Allen et al. 2002; Hollerhage et al. 2014). These mice demonstrated significant motor incoordination, reduced long-term and short-term spatial memory and recognition memory when compared to control group (Takeuchi et al. 2011; Sun et al. 2020; Watt et al. 2020; Di et al. 2021; Onishi et al. 2021; Zampar and Wirths 2021) and showed abnormal behaviors resembling deficiencies in human tauopathies (Takeuchi et al. 2011). These behavioural deficits were proved in our study which was reversed by AMI treatment. Interestingly, AMI treatment improved recognition memory and spatial memory in transgenic mouse model of AD previously (Chadwick et al. 2011; Lin et al. 2022).

It is important to highlight that the rationale for selecting amitriptyline in this study was based on its classification as a FIASMA rather than on its well-known antidepressant or anticholinergic properties. Our experimental design specifically utilized the colonic ASM-inhibitory effect of amitriptyline to investigate its mechanistic role in modulation of gut dysbiosis, colonic inflammation, systemic inflammation and tau pathology through bidirectional gut brain axis. Notably, this approach is mechanistically distinct from the epidemiological associations between long-term anticholinergic antidepressant use and cognitive decline in humans, these effects are not the focus of our investigation. Thus, the findings presented here should not be interpreted in the context of amitriptyline’s psychiatric indications, but rather as a promising evidence for therapeutic colonic ASM modulation in management of tauopathy.

## Limitations

This study was primarily designed to investigate the role of FIASMA in management of tauopathy by inhibition of colonic ASM and focused on the relationship between colonic ASM activity, colonic ceramide levels, GM diversity, dysbiosis, intestinal wall permeability, systemic inflammation and tau pathology. While our findings highlight important mechanistic links in usage of amitriptyline – as one of FIASMA drugs- as a neuroprotective against tau-driven neurodegeneration. Regarding AD – the most common tauopathy, key limitation of this study is that we did not test this mechanistic pathway in models of β-amyloid pathology. Because AD involves both tau and β-amyloid proteins aggregation in brains or β -amyloid proteins in gut as reported recently, it remains unclear whether inhibition of colonic ASM by FIASMA contributes similarly to β-amyloid aggregation, oligomerization, or early pathogenic events. Future studies using β-amyloid–based models, as well as combined tau–amyloid models, will be necessary to fully determine whether inhibition of colonic ASM by FIASMA is a shared neuroprotective tool across multiple pathological hallmarks of AD.

## Conclusion

The current data suggests that AMI exerts its neuroprotective effects against tauopathy through modulation of colonic ASM activity, associated gut ceramide levels, GM, colonic inflammation and membrane integrity through bidirectional gut-brain axis leading to distinct reduction in P-tau in hippocampus mainly by upregulation of PP2A which regulates tau aggregation and improves neurogenesis. Further, AMI improves recognition, short-term and long-term spatial memory and decreases motor incoordination in P301S mice suggesting that targeting colonic ASM is a promising therapeutic intervention in management of tauopathy.

## Supplementary Information

Below is the link to the electronic supplementary material.

Supplementary Material 1 (DOCX 580 KB)

Supplementary Material 2 (DOCX 1.04 MB)

Supplementary Material 3 (DOCX 729 KB)

## Data Availability

The datasets generated during and/or analyzed during the current study are available from the corresponding author on reasonable request. Sequence datasets generated for this study are deposited at NCBI sequence-reads archive (SRA), under project # PRJNA1203910.

## Abbreviations

AD: Alzheimer’s disease
AMI: Amitriptyline
ASM: Acid sphingomyelinase
BBB: Blood brain barrier
BDNF: Brain-derived neurotrophic factor
DI: Discrimination index
ELISA: Enzyme-linked immunosorbent assay
FIASMA: Functional inhibitor of acid sphingomyelinase
FTLD: Frontotemporal lobar degeneration
GM: Gut microbiota
H&E: Hematoxylin and Eosin
IL-22: Interleukin-22
LEfSe: Linear Discriminant Analysis Effect Size
LPS: Lipopolysaccharide
MWM: Morris water maze
MYD88: Myeloid Differentiation Factor 88
NF-KB: Nuclear Factor Kappa B
NFTs: Neurofibrillary tangles
NOR: Novel object recognition
OTUs: Operational taxonomic units
PBS: Phosphate buffered saline
PERMANOVA: Permutational Multivariate Analysis of Variance
PP2A: Protein phosphatase A2
PSP: Progressive supranuclear palsy
P-tau: Phosphorylated tau
SD: Standard deviation
SPL: Sphingolipids
TBST: Tris-buffered saline with Tween 20
TLR4: Toll like receptor 4
TNF-α: Tumor necrosis factor alpha
ZO-1: Zonula Occludens-1
