# Supplementary material for: Gut Microbiome–Sphingolipid Metabolism–Brain Axis Interactions: Neuroprotective Effects of Amitriptyline as Functional Inhibitor of Acid Sphingomyelinase in a Mouse Model of Tauopathy
Source: J Neuroimmune Pharmacol. 2026 Jan 3;21(1):3. doi: 10.1007/s11481-025-10270-x (PMC12764700; doi:10.1007/s11481-025-10270-x)
Supplement: Supplementary file 1 — Supplementary Material 1 (DOCX 580 KB) [file 11481_2025_10270_MOESM1_ESM.docx]

## Supplementary Figures

# Title:

Gut microbiome–sphingolipid metabolism–brain axis interactions: neuroprotective effects of amitriptyline as functional inhibitor of acid sphingomyelinase in a mouse model of tauopathy

# Journal:

Journal of neuroimmune pharmacology

**Authors:**

Mennatallah O. Zaki**^a^**, Asmaa M. Khalil**^b^**, Heba Attia**^c^**, Saleh Alseekh**^d^**, Ahmed F. Mohamed**^e,f*^**, Mohammed F. EL-Yamany**^e^**

**^a^**: Department of Pharmacology and Toxicology, Faculty of Pharmacy, Horus University, New Damietta, Egypt

**^b^**: Department of Pharmacognosy, Faculty of Pharmacy, Cairo University, Cairo, 11562, Egypt

**^c^**: Department of Microbiology and Immunology, Faculty of Pharmacy, Cairo University, 11562 Cairo, Egypt

**^d^**: Max Planck Institute of Molecular Plant Physiology, Potsdam-Golm, Germany

**^e^**: Department of Pharmacology and Toxicology, Faculty of Pharmacy, Cairo University, 11562 Cairo, Egypt

^f^: Pharmacology and Toxicology Department, Faculty of Pharmacy, King Salman International University (KSIU), South Sinai, 46612, Egypt

* Correspondence: All correspondence should be addressed to:

**Ahmed F. Mohamed**

Pharmacology and Toxicology Department, Faculty of Pharmacy, Cairo University, Cairo 11566, Egypt

Phone: +201220069121

E-mail address: [ahmed.fathi@pharma.cu.edu.eg](mailto:ahmed.fathi@pharma.cu.edu.eg)

ORCID: 0000-0002-7678-0643


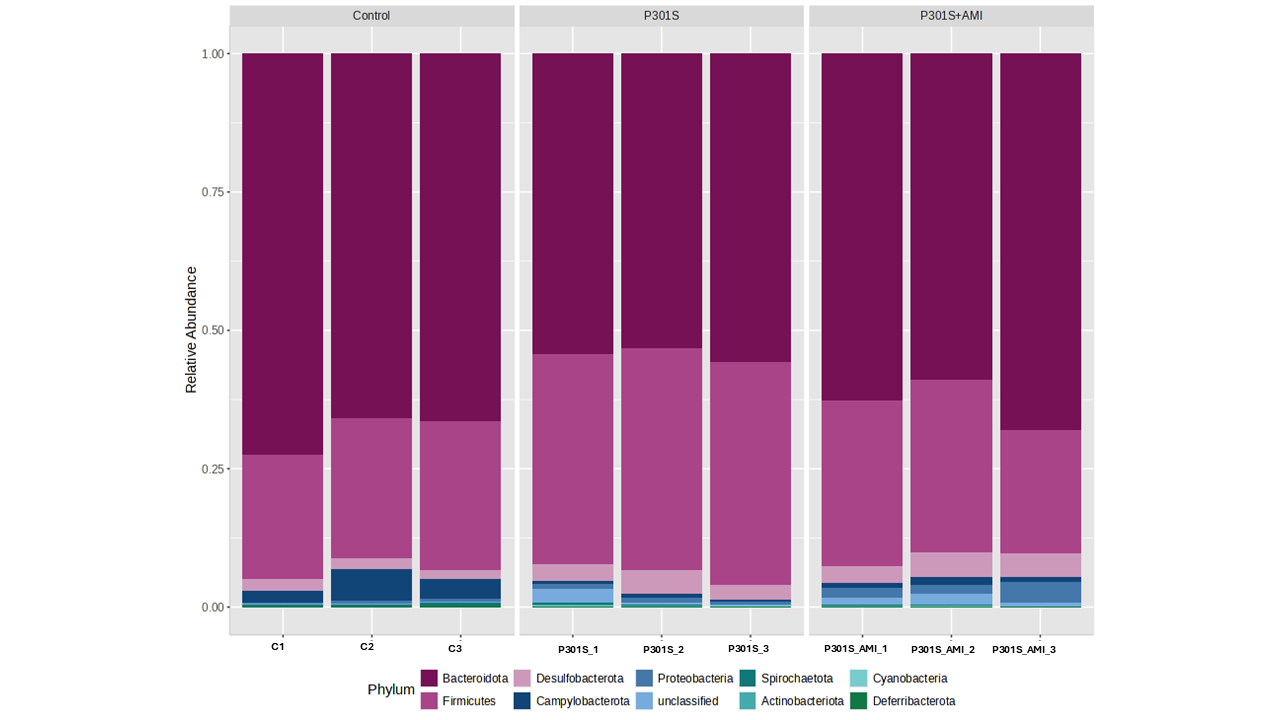


Fig. supplementary 1: Taxonomic composition of the fecal microbiome across the three experimental groups. Summary of the relative abundance of different taxonomic units detected in the fecal samples at the phylum levels.


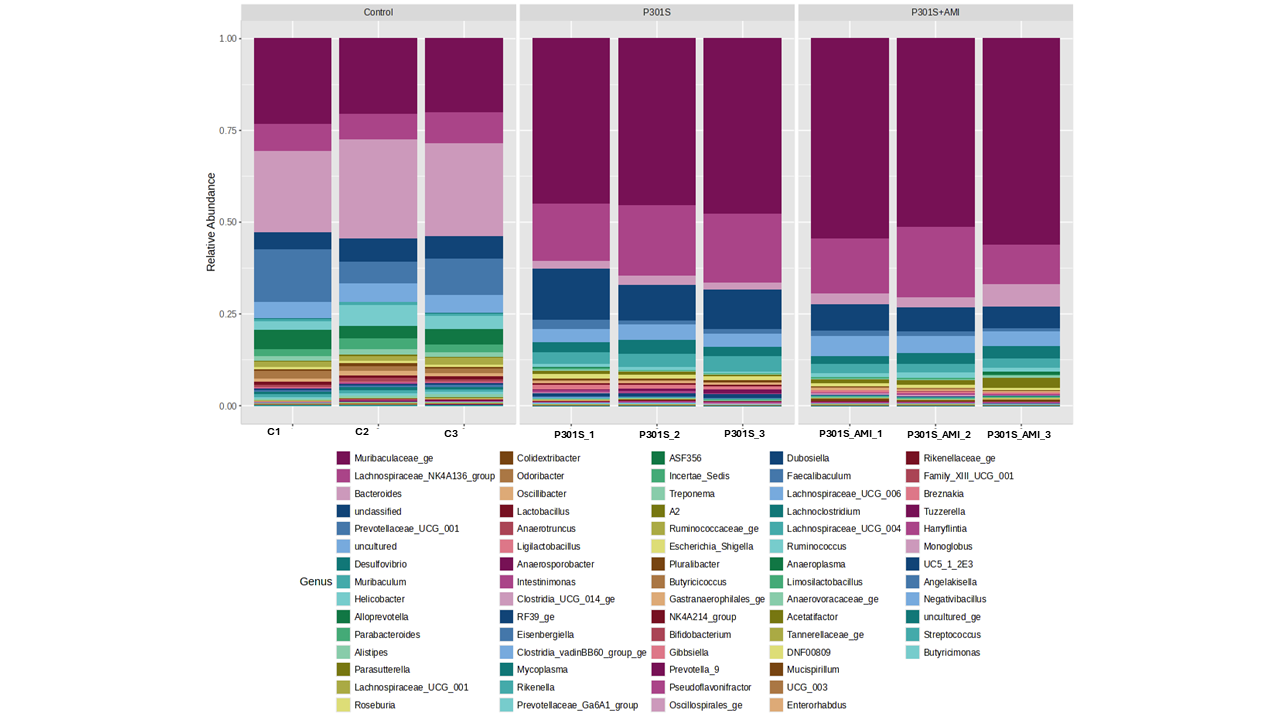


Fig. supplementary 2: Taxonomic composition of the fecal microbiome across the three experimental groups. Summary of the relative abundance of different taxonomic units detected in the fecal samples at the genus levels.
