# Supplementary material for: Gut Microbiome–Sphingolipid Metabolism–Brain Axis Interactions: Neuroprotective Effects of Amitriptyline as Functional Inhibitor of Acid Sphingomyelinase in a Mouse Model of Tauopathy
Source: J Neuroimmune Pharmacol. 2026 Jan 3;21(1):3. doi: 10.1007/s11481-025-10270-x (PMC12764700; doi:10.1007/s11481-025-10270-x)
Supplement: Supplementary file 2 — Supplementary Material 2 (DOCX 1.04 MB) [file 11481_2025_10270_MOESM2_ESM.docx]

## Supplementary Figures

# Title:

Gut microbiome–sphingolipid metabolism–brain axis interactions: neuroprotective effects of amitriptyline as functional inhibitor of acid sphingomyelinase in a mouse model of tauopathy

# Journal:

Journal of neuroimmune pharmacology

**Authors:**

Mennatallah O. Zaki^a^, Asmaa M. Khalil^b^, Heba Attia^c,d^, Saleh Alseekh^e^, Ahmed F. Mohamed^f,g*^, Mohammed F. EL-Yamany^f^

^a^: Department of Pharmacology and Toxicology, Faculty of Pharmacy, Horus University, New Damietta, Egypt

^b^: Department of Pharmacognosy, Faculty of Pharmacy, Cairo University, Cairo, 11562, Egypt

^c^: Department of Microbiology and Immunology, Faculty of Pharmacy, Cairo University, 11562 Cairo, Egypt

^d^: Center for Genome and Microbiome Research, Cairo University, 11562 Cairo, Egypt

^e^: Max Planck Institute of Molecular Plant Physiology, Potsdam-Golm, Germany

^f^: Department of Pharmacology and Toxicology, Faculty of Pharmacy, Cairo University, 11562 Cairo, Egypt

^g^: Pharmacology and Toxicology Department, Faculty of Pharmacy, King Salman International University (KSIU), South Sinai, 46612, Egypt

* Correspondence: All correspondence should be addressed to:

**Ahmed F. Mohamed**

Pharmacology and Toxicology Department, Faculty of Pharmacy, Cairo University, Cairo 11566, Egypt

Phone: +201220069121

E-mail address: [ahmed.fathi@pharma.cu.edu.eg](mailto:ahmed.fathi@pharma.cu.edu.eg)

ORCID: 0000-0002-7678-0643


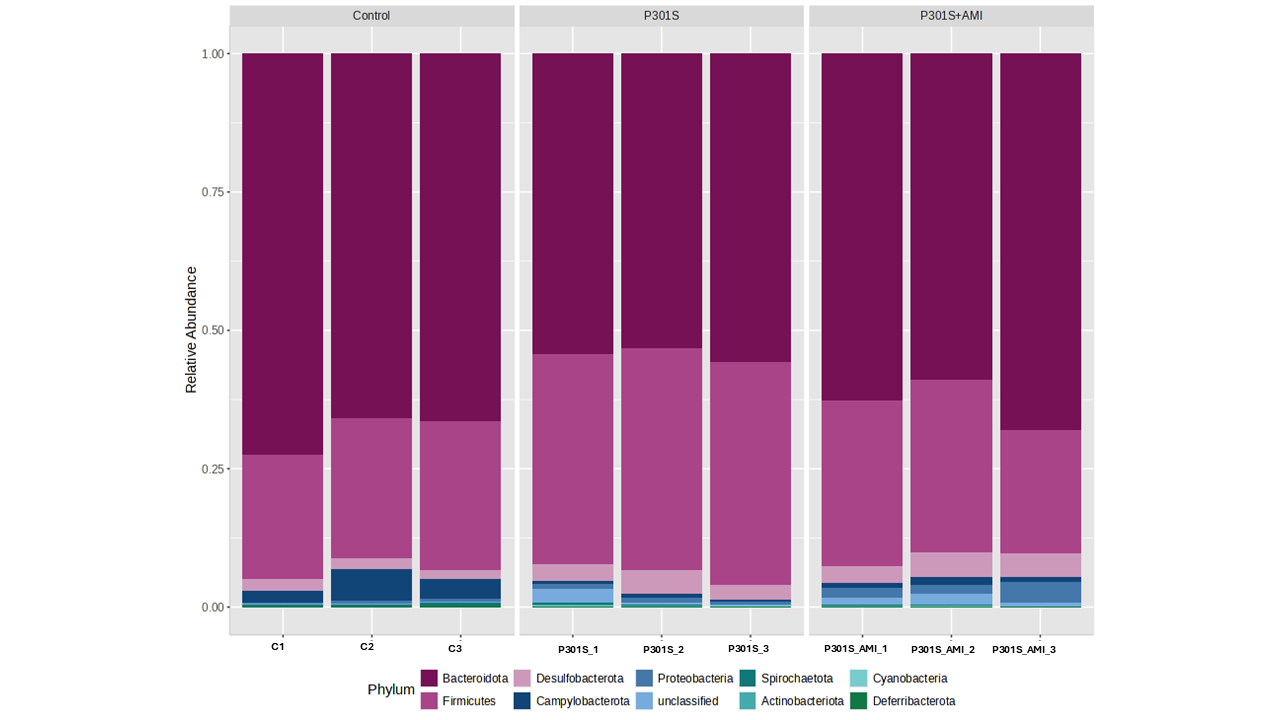


Fig. supplementary 1: Taxonomic composition of the fecal microbiome across the three experimental groups. Summary of the relative abundance of different taxonomic units detected in the fecal samples at the phylum levels.


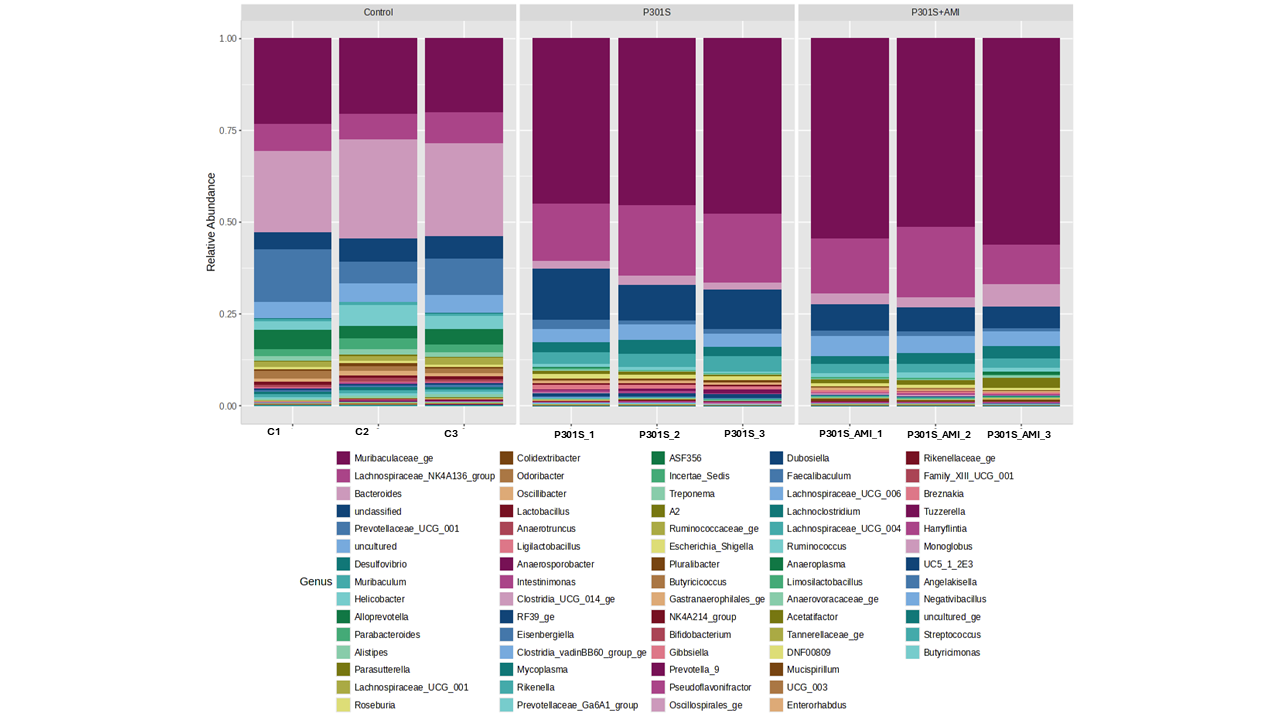


Fig. supplementary 2: Taxonomic composition of the fecal microbiome across the three experimental groups. Summary of the relative abundance of different taxonomic units detected in the fecal samples at the genus levels.

| 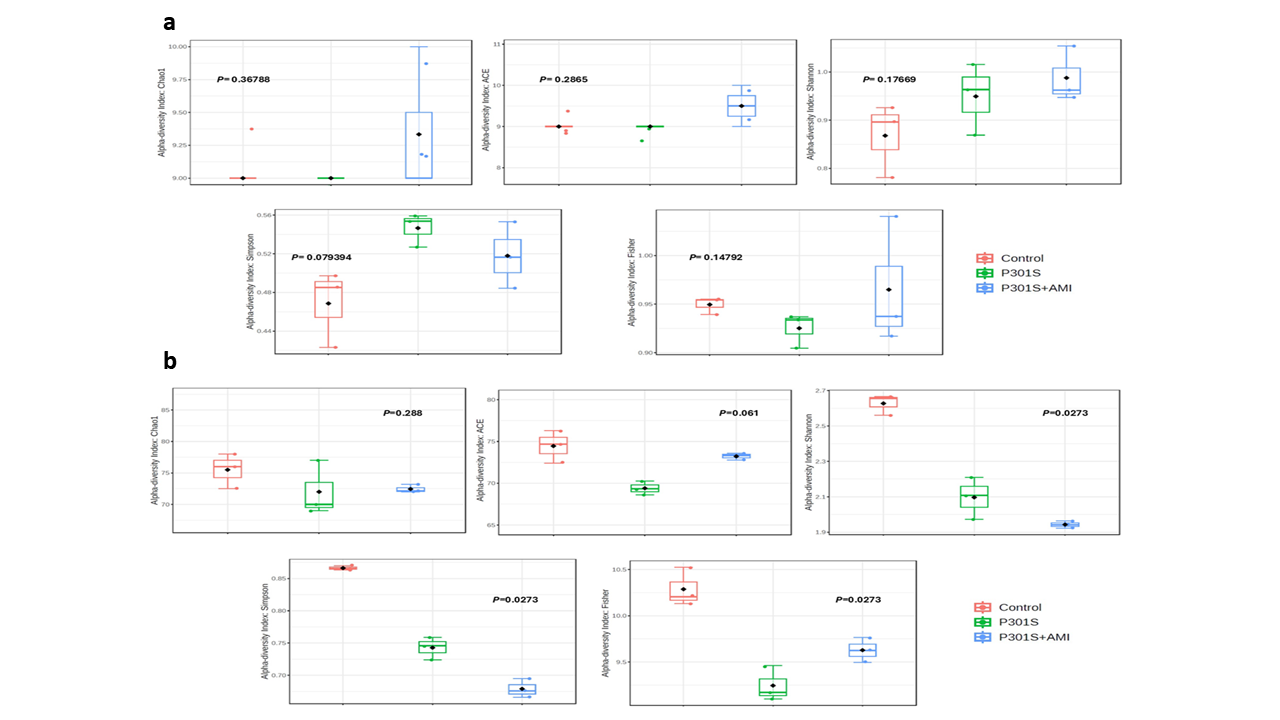 |
| --- |
| **Fig. supplementary 3: Different alpha diversity metrics (Chao1, ACE, Shannon, Simpson, and Fisher diversity indices), for microbiome samples, at different treatment groups, (a) at phylum level and (b) at genus level.**  Alpha diversity profiling and significance testing were conducted using Kruskal Wallis test. The level of significance was fixed at a *p*-value of ≤0.05. |

| 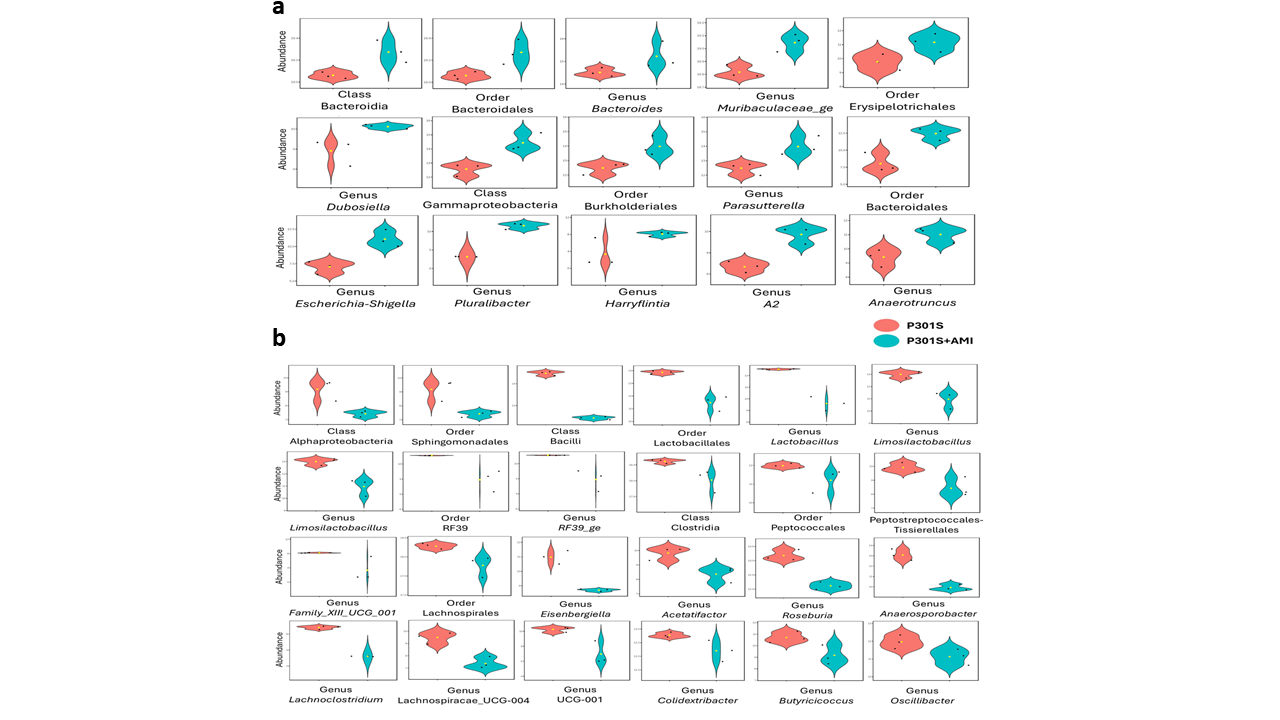 |
| --- |
| **Fig. supplementary 4: Taxa with significant differences in relative abundance in P301S mice and AMI treated P301S mice. Violin plots describe differential relative abundance of significant taxa in different samples; (a) abundant taxa in AMI treated P301S mice group, and (b) scarce taxa in the AMI treated P301S mice group.**  The significance was identified by LEfSe analysis, with *p* value cutoff of 0.05, and *p*value adjustment by the FDR method. Y-axis: Relative abundance expressed as log-transformed counts. |
