# Supplementary figures and images for: Gut Microbiome–Sphingolipid Metabolism–Brain Axis Interactions: Neuroprotective Effects of Amitriptyline as Functional Inhibitor of Acid Sphingomyelinase in a Mouse Model of Tauopathy
Source: J Neuroimmune Pharmacol. 2026 Jan 3;21(1):3. doi: 10.1007/s11481-025-10270-x (PMC12764700; doi:10.1007/s11481-025-10270-x)

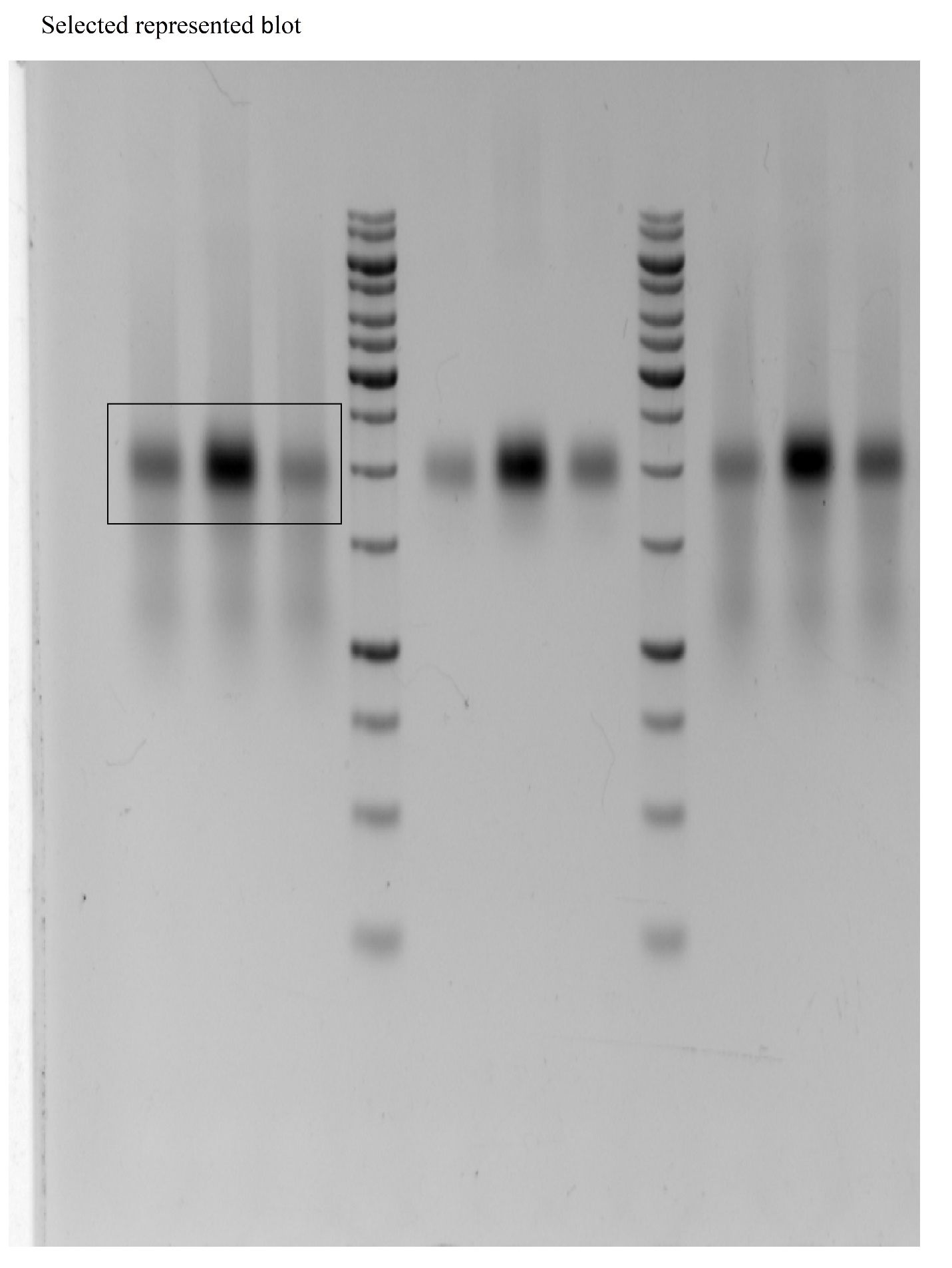


Tau protein


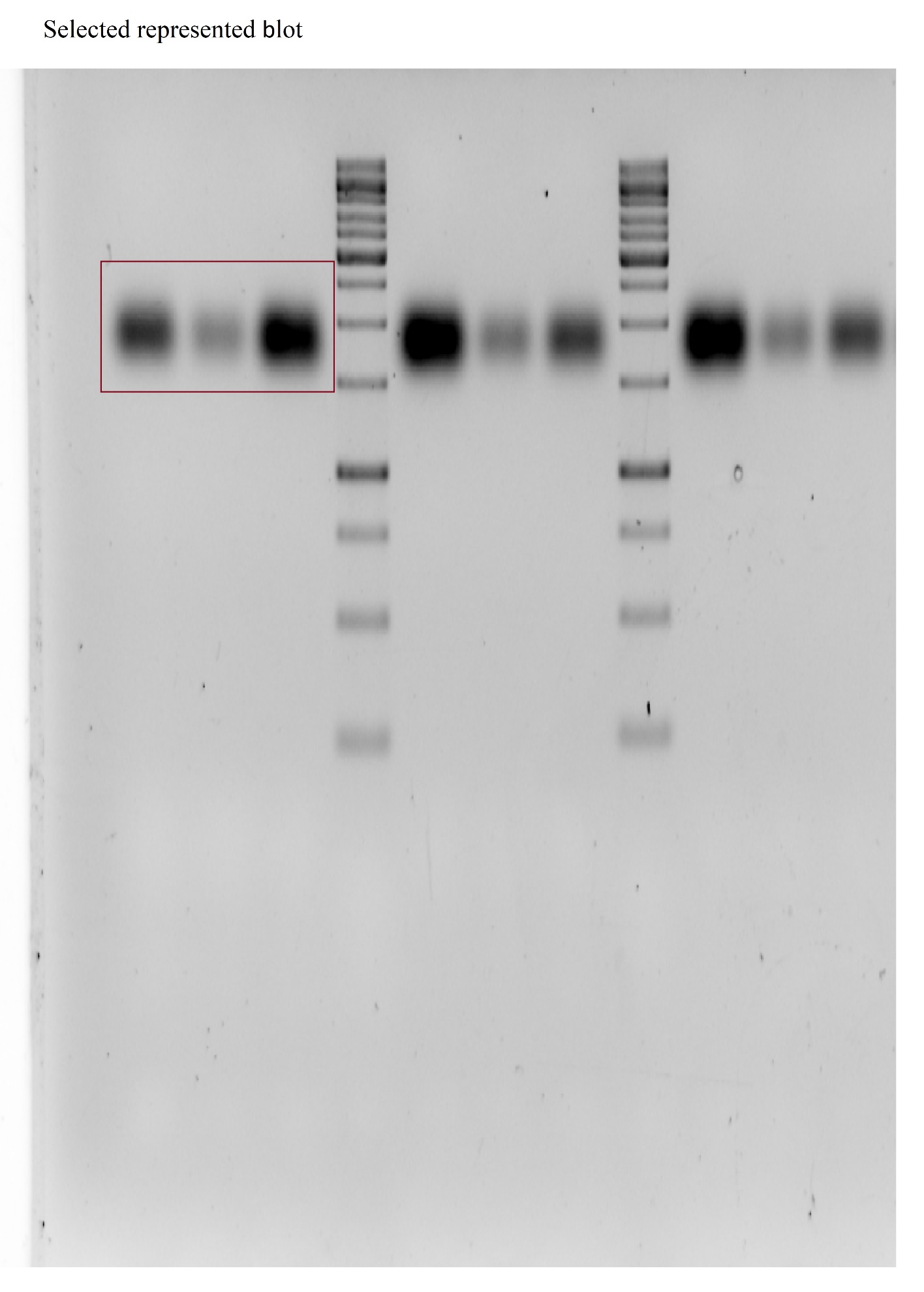


Protein phosphatase 2A


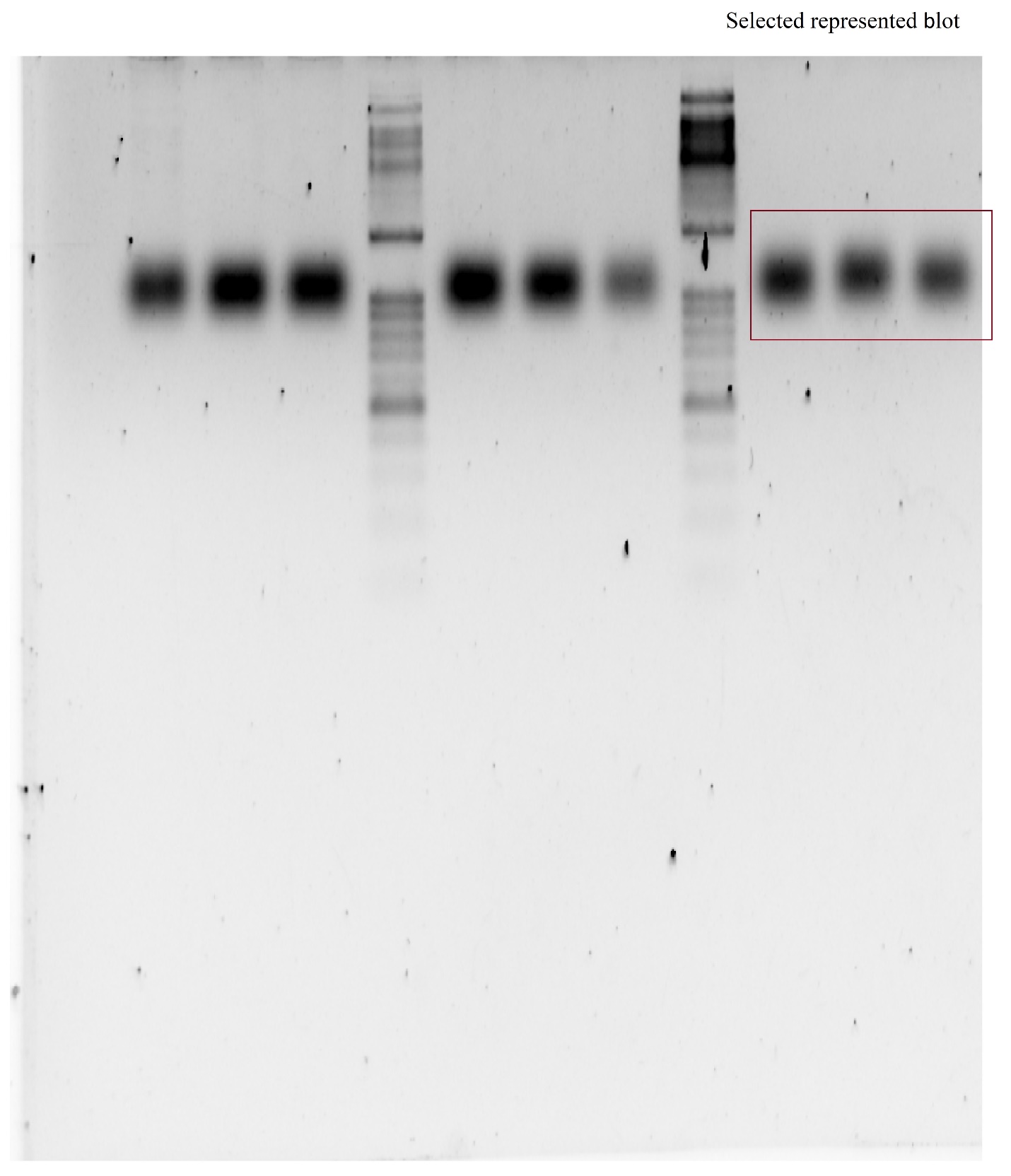


β-actin

Supplement: Supplementary file 3 — Supplementary Material 3 (DOCX 729 KB) [file 11481_2025_10270_MOESM3_ESM.docx]
